# *AtZAT10/STZ1* improves drought tolerance and increases fiber yield in cotton

**DOI:** 10.3389/fpls.2024.1464828

**Published:** 2024-10-21

**Authors:** Lixia Qin, Hehe He, Liqun Yang, Huanyang Zhang, Jing Li, Yonghong Zhu, Jianguo Xu, Gaili Jiao, Chengbin Xiang, Chuangyun Wang, Shenjie Wu

**Affiliations:** ^1^ Shanxi Agricultural University, Taiyuan, China; ^2^ Shanxi Province Rural Industry Integration Development Center, Taiyuan, China; ^3^ School of Life Sciences, University of Science and Technology of China, Hefei, China

**Keywords:** AtZAT10/STZ1, drought stress, transgenic cotton, fiber yield, transcriptomic analysis

## Abstract

Drought poses a significant challenge to global crop productivity, necessitating innovative approaches to bolster plant resilience. Leveraging transgenic technology to bolster drought tolerance in crops emerges as a promising strategy for addressing the demands of a rapidly growing global populace. AtZAT10/STZ1, a C2H2-type zinc finger protein transcription factor has shown to significantly improve Arabidopsis’ tolerance to various abiotic stresses. In this study, we reports that *AtSTZ1* confers notable drought resistance in upland cotton (*Gossypium hirsutum*), amplifying cotton fiber yield under varying conditions, including irrigated and water-limited environments, in field trials. Notably, *AtSTZ1*-overexpressing transgenic cotton showcases enhanced drought resilience across critical growth stages, including seed germination, seedling establishment, and reproductive phases. Morphological analysis reveals an expanded root system characterized by an elongated taproot system, increased lateral roots, augmented root biomass, and enlarged cell dimensions from transgenic cotton plants. Additionally, higher contents of proline, chlorophyll, soluble sugars, and enhanced ROS-scavenging enzyme activities are observed in leaves of transgenic plants subjected to drought, underscoring improved physiological adaptations. Furthermore, transgenic lines exhibit heightened photosynthetic rate, increased water use efficiency, and larger stomatal and epidermal cell sizes, coupled with a decline in leaf stomatal conductance and density, as well as diminished transpiration rates compared to the wild type counterparts. Transcriptome profiling unveils 106 differentially expressed genes in transgenic cotton leaves post-drought treatment, including protein kinases, transcription factors, aquaporins, and heat shock proteins, indicative of an orchestrated stress response. Collectively, these findings underscore the capacity of *AtSTZ1* to augment the expression of abiotic stress-related genes in cotton following drought conditions, thus presenting a compelling candidate for genetic manipulation aimed at enhancing crop resilience.

## 1 Introduction

Cotton, an important natural fiber cash crop, is extensively cultivated worldwide. However, the yield and quality of cotton are mainly limited by drought, as a primary environmental factor (Rocha-Munive et al., 2018; Anwar et al., 2022), necessitating improvements in drought resistance. Traditional breeding methods, while successful in improving the resilience of plants against abiotic stress factors, are costly, time-consuming, and labor-intensive (Varshney et al., 2011). Consequently, the utilization of genetic engineering technology to develop drought-resistant and water-saving cotton varieties has emerged as a new trend in the field of cotton breeding (Mahmood et al., 2019). Plants have evolved intricate regulatory systems for controlling the expression of specialized genes to acclimatize to drought stress (Gupta et al., 2020). Roots and leaves of plants have independently evolved synchronized defense mechanisms responding to drought stress (Kim et al., 2020). The physiological and morphological features of plant roots and leaves affect crop growth, development, overall yield and quality (Wasson et al., 2012; Uga et al., 2013). Under drought stress, root diameter, depth and density have a positive correlation with plant vigor (Wasson et al., 2012), and stomatal density exhibits a positive association with resilience to drought stress (Clauw et al., 2016; Qi et al., 2018). Therefore, identifying stress-resistant genes for cotton breeding is desirable, and altering the root and leaf structure of cotton through transgenic technology holds significant potential for breeding drought-resistant cotton varieties.

Zinc finger proteins (ZFPs) belong to a class of transcription factor characterized by a highly conserved fingerlike domain. These domains form a stable three-dimensional structure facilitated by zinc ions surrounded by different numbers of histidine (His) and L (+)-cysteine (Cys) residues, including an anti-parallel β-fold and an α-helix (Razin et al., 2012; Hu et al., 2019). Currently, there are ten types, such as Cys2/His2 (C2H2), C2HC, C6, C8, C2HC5, and C3HC4, as the known structures and classifications of zinc finger proteins. Among these, the C2H2 ZFPs, also called as typical TFIIII ZFPs, constitute a sizable family among eukaryotic transcription factors. In plants, the C2H2 zinc finger domain is the most crucial DNA binding element and primarily participates in protein−RNA, protein−DNA, and protein−protein interactions (Wolfe et al., 2000; Pabo et al., 2001; Fedotova et al., 2017). This domain contains approximately thirty amino acid residues, featuring a consensus motif of CX2-4CX3FX5LX2HX3-5H. Among these amino acids, one Zn^2+^ links two histidine- and two cysteine- residues in a tetrahedral form to sustain protein stability (Pabo et al., 2001). In rice and *Arabidopsis thaliana*, 189 and 176 ZFPs, respectively, have been isolated and identified (Ciftci-Yilmaz and Mittler, 2008; Agarwal et al., 2007).

Previous researches have established that C2H2 ZFPs participated in response to multiple abiotic stresses (Sun et al., 2019; Zhang et al., 2014). In rice, the C2H2 transcription factor ZFP182 plays a role in the antioxidant defense system mediated by ABA (Zhang et al., 2012). Overexpression of *OsDRZ1* in rice boosts drought resilience in seedlings, resulting in lower levels of reactive oxygen species (ROS) and increased free proline content. Conversely, *OsDRZ1*-silenced rice plants exhibited decreased antioxidant activity and enhanced sensitivity to drought, indicating that *OsDRZ1* modulates drought stress by acting as a transcription inhibitor. Notably, the overexpression of *OsDRZ1* in rice does not inhibit but promotes plant growth (Yuan et al., 2018). Two C2H2 ZFPs from soybean, GmZFP1 and GmZF1, have been confirmed to increase drought and cold tolerance in transgenic *Arabidopsis* (Yu et al., 2014; Huang et al., 2006). In *Arabidopsis*, the expression of *GmZFP3* improves resilience to PEG and ABA while decreasing the sensitivity to drought (Zhang et al., 2016). *ZFP182* is triggered by ABA, drought and cold, and heterologous expression of *ZFP182* gene in rice and tobacco enhances plant resilience to high salinity stress (Huang et al., 2006). In *Arabidopsis*, the transcription level of *ZAT18* is increased under dehydration stress, and multiple stress response genes, including *ERD7*, *RAS1*, *LEA6* and *COR47*, as well as hormone signal-responsive genes, such as *PYL5* and *JAZ7*, have been recognized as target genes of ZAT18. *Arabidopsis* plants overexpressing *ZAT18* exhibit increased tolerance to drought-stress, while *ZAT18* mutant lines are more sensitive. Compared to the wild counterparts, *ZAT18*-overexpressing plants exhibit decreased water loss and higher antioxidant enzymes activity in leaves following drought treatment (Yin et al., 2017). In *Arabidopsis*, *ZAT12* plays a pivotal role in responding to abiotic stress and ROS, modulating the plant’s adaptive mechanisms to strong light and oxidative stress, and enhancing antioxidant activity and adaptability to drought stress (Lee et al., 2016; Rai et al., 2013; Davletova et al., 2005). *ZAT12* upregulates the expression of the *WRKY25*, *ZAT7* and *APX1* genes related to ROS signal transduction. Under abiotic stress, co-expression of *ZAT12* and *APX1* regulates ascorbic acid metabolism. *ZAT12* also acts as a crucial component of ROS signal transduction and interacts with other genes to mitigate ROS damage (Wang et al., 2020).

*Arabidopsis ZAT10* (SALT TOLERANCE ZINC FINGER, STZ) possesses two C2H2 domains, and its transcriptional level is increased in response to diversified conditions including ABA, drought, high salinity, and low temperature. The overexpression or inhibition of *ZAT10* in *Arabidopsis* can enhance plant resilience against a range of abiotic stresses, including antioxidation, anti-osmotic stress, anti-heating, and anti-salt stress, positioning ZAT10 as a bidirectional regulator in abiotic stresses (Mittler et al., 2006). The ERA domain at the C-terminus of *ZAT10* functions as an inhibitor and is regulated by MPK3 and MPK6 phosphorylation (Nguyen et al., 2016). Sakamoto et al. (2004) reported that the overexpression of the *STZ* gene could enhance *Arabidopsis*’s drought tolerance. In contrast, Woo et al. (2008) exhibited that the drought resistance of transgenic *Arabidopsis* downregulated expressing *STZ* via RNA interference was significantly improved. Recent studies have indicated that *MhZAT10* is essential for apple plants to cope with cold and drought stress. The heterologous overexpression of *MhZAT10* in the cold-sensitive variety ‘G935’ promotes overwintering sprouting, while the expression inhibition of *MhZAT10* in the frost-resistant variety ‘SH6’ reduces stress tolerance. MhDREB2A directly regulates and activates the expression of *MhZAT10* responding to drought, and overexpression of *MhDREB2A* and *MhZAT10* increases the plant’s resilience to cold and drought. However, the overexpression of *MhDREB2A* and the inhibition of *MhZAT10* decrease the resistance of plants to drought and cold. Furthermore, MhZAT10 has been identified as an upstream regulator of several downstream target genes, including MhWRKY31, which exhibits drought tolerance, and MhMYB88 and MhMYB124, both demonstrating cold tolerance (Li et al., 2023).

At present, although most studies on C2H2 ZFPs have concentrated on fundamental stresses, comprising high salt, low temperature and drought treatments, the vital function of these proteins in affecting crop quality and yield has rarely been reported. Some C2H2 ZFPs participate in and integrate various stress tolerance signaling pathways, but their specific regulatory mechanisms remain unclear (Huang et al., 2019). Previous studies have revealed that when overexpressed in *Arabidopsis*, the cotton C2H2 ZFPs GhDi19-1 and GhDi19-2 result in an increased susceptibility of the plants to ABA and high salt stress (Li et al., 2010). More lately, Fan et al. (2021) cloned the *G. hirsutum* gene *GhZAT67* and found that *GhZAT67*-silenced plants via virus-induced gene silencing (VIGS) wilted more than wild-type plants following alkaline treatment, indicating *GhZAT67* participates in the response to alkaline stress. Additionally, *GhCSTZ* was expressed in cotton seedlings following salt stress treatment (An et al., 2016). The expression of GhZAT10 in roots was higher than that in stems and leaves, and up-regulated after chilling stress in these tissues. The GhZAT10-silenced cotton plants were more sensitive to low temperature but not to drought than wild type, and GhZAT10 plays a positive role to response the chilling stress in upland cotton (Yang et al., 2020), moreover, GhZAT10 and GhCSTZ did not affect fiber yield.

However, the role of *AtZAT10/AtSTZ1* in drought resistance, yield and quality has not been reported in cotton. In the current research, our primary objective was to obtain *AtSTZ1*-overexpressing transgenic cotton plants and evaluate their drought resistance in the laboratory conditions, greenhouse environments, and in actual field settings. The results revealed that overexpression of *AtSTZ1* in cotton greatly enhanced drought resistance and led to improved fiber yield in the field. Our results suggests that *AtSTZ1* holds promising potential as a target gene for crop improvement with drought tolerance.

## 2 Materials and methods

### 2.1 Vector construction and genetic transformation

Construction of the *AtSTZ1* overexpression vector was undertaken following previously established methods (Yu et al., 2016). Briefly, the complete *AtSTZ1* cDNA sequence was inserted into the binary vector pCB3000, containing the CaMV 35S promoter. Subsequently, the recombinant plasmid (**Supplementary Figure S1A**) was introduced into hypocotyl explants of upland cotton (*Gossypium hirsutum*) cultivars Zhongmian 44 and R15 (Qin et al., 2017). Positive transgenic plants from T_0_ - T_3_ generations were selected and determined according to Qin et al. (2017). The primers utilized for vector construction can be found in **Supplementary Table S1**.

### 2.2 qRT-PCR analysis

Extraction and purification of total RNA from the transgenic cotton leaves and synthesis of first-strand cDNA are referred to Li et al. (2005). As mentioned earlier, quantitative real-time PCR (qRT-PCR) analysis was utilized to detect the transcriptional level of *AtSTZ1* and additional genes that are associated with drought stress. This analysis was conducted in accordance with the methodology described by Li et al. (2005). The *GhHis3* gene (AF024716) served as the internal reference for gene expression normalization. The specific primers utilized for quantitative reverse transcription polymerase chain reaction (qRT-PCR) are detailed in **Supplementary Table S1**.

### 2.3 Drought tolerance trials

The seed germination trials involved sowing seeds from homozygous *AtSTZ1*-overexpressing transgenic lines (OE-4, -6, generated from R15 as explants, and OE-7 and -9 generated from Zhongmian 44 as explants) and the wild type on 10% PEG6000-saturated filter papers. These seeds were germinated in an artificial light incubator with 16-hour light/8-hour dark cycles at 28°C. The germination rate was determined referring to Qin et al. (2014).

Drought tolerance tests were conducted on seedlings from both the wild-type and transgenic cotton in a greenhouse according to Yu et al. (2016). The same author’s method was used to assess the drought resistance and agronomic performance of transgenic cotton plants in the field. These field experiments were performed in Yuncheng City, Shanxi Province, China and Shihezi City, Xinjiang Province, China. Images were taken when plants exhibited noticeable drought stress phenotypes during seedling development, the reproductive period and the mature stage (Yu et al., 2016).

The growth status of the seedlings was recorded on day 15, and a lateral root count was calculated. After 25 days of drought stress, the primary root length of early plants was measured. The roots were cut from AtSTZ1-overexpressing transgenic cotton plants treated with drought for 15 days and 25 days, respectively. Samples of leaves and primary roots were collected from plants that had undergone a 25-day drought treatment. The root and leaf surface imprint methods, referring to Yu et al. (2008), were used. The number of stomata and root cells were calculated, and their sizes were measured referring to the methods of Yu et al. (2016).

All experiments were independently replicated three times.

### 2.4 Determination of the water use efficiency, transpiration rate, and photosynthetic rate

The water use efficiency (WUE), transpiration rate, and photosynthetic rate in the leaves from 25-day-old transgenic cotton plants were determinated according to Yu et al. (2013). The WUE, transpiration rate and photosynthetic rate were assessed utilizing a photosynthesis measuring instrument (CIRAS-3, Portable photosynthesis System, Lufthansa Scientific Instruments Co., Ltd.).

### 2.5 Determination of MDA, free proline, soluble sugar, and chlorophyll contents and electrolyte leakage

Leaves from plants subjected to drought for 25 days or from normal control plants were utilized for the analysis of MDA, free proline, soluble sugar, and chlorophyll contents and electrolyte leakage. Proline and chlorophyll levels were quantified following the methodology outlined by Qin et al. (2014), while soluble sugars were determinated as per the protocol by Dubois et al. (1956). As previously mentioned, the MDA content was detected according to the procedure of the thiobarbituric acid (TBA) test (Cao et al., 2009), and electrolyte leakage was assessed referring to Bajji et al. (2002).

### 2.6 Measurement of POD, CAT, and SOD activities

The assays of POD, SOD, and CAT activities were performed following the protocol outlined by Ruan et al. (2011). Total protein content was determined by utilizing a Bradford Protein Assay Kit (Solarbio Science &Technology Co., Ltd, Beijing, China).

### 2.7 Transcriptomic analysis

Fourth true leaf tissues from both transgenic cotton lines and the wild-type controls following drought treatment for 25 days were collected, and extraction and purification of total RNA from the fourth true leaf were conducted, following the methodology outlined by Qin et al. (2017). Subsequently, RNA-seq libraries were constructed and sequenced by Nanjing Personal Gene Technology Co., Ltd. (Nanjing, Jiangsu, China, http://www.personalbio.cn). Details regarding the sequencing location/facility, read length, and type have been previously described by Zhou et al. (2020). Gene function was predicted and the distribution frequency of functional categories was calculated by gene ontology (GO) analysis.

Edger software was used for the analysis of differential gene expression derived from transcriptome sequencing data. The determination of differential gene expression was founded on fold change (FC ≥ 2 or FC ≤ -2) and P value (P ≤ 0.01). The Cutadapt (v2.7) software and parameters utilized for GO and heat map analyses refer to Zhang et al. (2021). The statistical analysis incorporated a twofold alteration in the average of biological replicates, coupled with a stringent false discovery rate threshold of below 0.05 (P < 0.05), to ensure rigor and accuracy.

## 3 Results

### 3.1 *AtSTZ1* increases fiber yield and improves agronomic traits in the field

To explore the role of *AtSTZ1* in cotton yield, we obtained 21 transgenic cotton lines overexpressing *AtSTZ1* in the R15 and Zhongmian 44 wild-type backgrounds and verified them via kanamycin resistance and PCR analyses (**Supplementary Figure S1**). To investigate the function of AtSTZ1 in response to drought, we selected four overexpressing homozygous lines with higher AtSTZ1 expression levels (**Supplementary Figure S1B**, OE-4 and -6, generated from R15 as explants; OE-7 and -9, generated from Zhongmian 44 as explants) for subsequent analyses.

**Figure 1:**
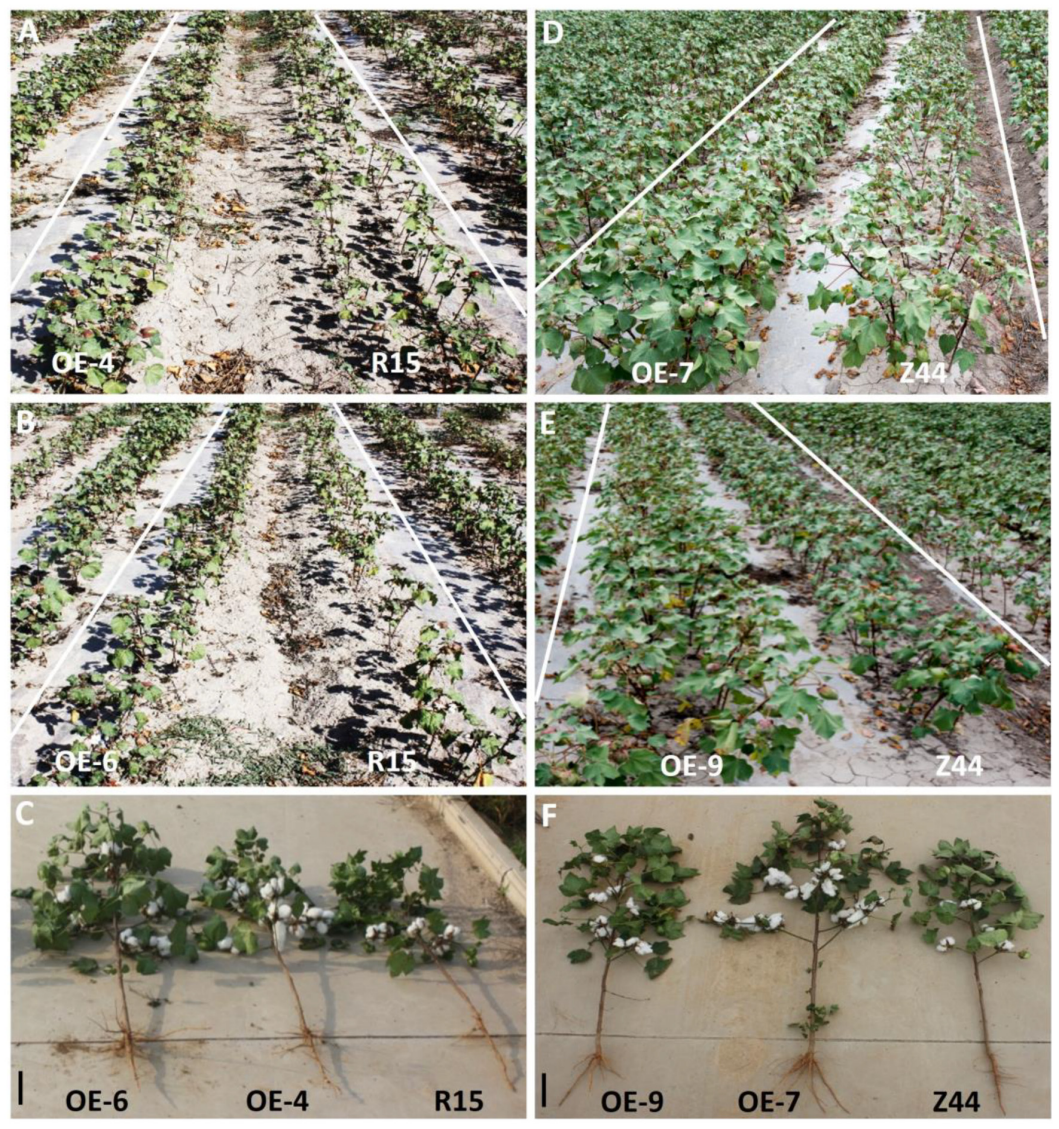
Significantly enhanced drought resistance of *AtSTZ1*-overexpressing transgenic cotton plants in the field. **(A, B, D, E)** Phenotypes of *AtSTZ1*-overexpressing transgenic cotton lines in Shihezi, Xinjiang, China. The plants were then photographed under natural drought conditions for 80 days. **(C, F)** Growth of *AtSTZ1* transgenic cotton lines during the mature growth stage in Yuncheng, Shanxi, China. OE-4, -6, *AtSTZ1-*overexpressing transgenic cotton lines 4 and 6 generated using R15 as explants; OE-7, -9: *AtSTZ1*-overexpressing transgenic cotton lines 7 and 9 generated using Zhongmian 44 as explants. Bars=8 cm.

To evaluate the transgenic lines’ performance in the field, considering both irrigated and natural drought conditions, we constructed field trials at two different field locations (Shihezi, Xinjiang and Yuncheng, Shanxi). A major field experiment was performed in Shihezi, Xinjiang Province, China, from April to September, a period characterized by scarce rainfall throughout the growing season. As illustrated in **Figures 1A, B, D, E**, compared to the wild type, noticeably better growth and improved drought resilience is observed in four transgenic lines (-4, -6, 7, and -9), and their agronomic trait data were collected along with those of the wild-type controls.

A comparison of fiber yield and agronomic traits between *AtSTZ1* transgenic lines and the wild- type counterparts was conducted under normal irrigation and natural non-irrigation conditions in the field. As indicated in **Table 1**, under adequate water conditions, relative to that of the wild-type controls (R15 and Zhongmian 44), the fruit branch number (P ≤ 0.02), boll number (P ≤ 0.03), plant height (P ≤ 0.03), and boll fresh weight (P ≤ 0.03) of the transgenic lines 4, 6, 7 and 9 significantly increased, and the yield of seed cotton per plant rose by 18.20%, 11.22%, 18.25%, and 14.24%, respectively (P ≤ 0.03), with a significant decrease in boll shedding (P ≤ 0.04). Notably, under natural drought stress, the *AtSTZ1* transgenic plants displayed better agronomic traits than did the wild-type plants (**Table 1**). The yield of cotton seed fiber from transgenic lines 4, 6, 7 and 9 increased markedly, by 21.88%, 20.82%, 25.81% and 23.04%, respectively (P ≤ 0.02), relative to that of the wild type controls. Besides, the fruit branch number (P ≤ 0.02), boll number (P < 0.01), boll fresh weight (P ≤ 0.02), and plant height (P < 0.01) have an obvious increase, while boll shedding per plant (P ≤ 0.03) decreased in the four transgenic lines in contrast to the wild type controls (**Table 1**). Importantly, the relative cotton fiber yield of the transgenic lines demonstrated a more significantly increase under natural arid environment than under normal, well-watered conditions (**Table 1**). Under natural drought condition, the field performance (including cotton fiber yield, boll fresh weight, boll number, fruit branch number, and plant height) of AtSTZ1 transgenic cotton lines was positively correlated with the expression level of AtSTZ1 (**Table 1**). Our findings conclusively demonstrated that the overexpression of *AtSTZ1* in cotton not only enhanced drought resistance and simultaneously improved fiber yield in the field.

**Table 1:** Agronomic characteristics and cotton fiber yields between transgenic lines and wild-types in the field under the conditions of natural drought and water irrigation.

Genotype	Plant height (cm)	Fruit branch number per plant	Boll number per plant	Boll shedding number per plant	Boll fresh weight (g)	Cotton fiber yield (g plant^−1^)
Normal						
R15	68.00 ± 3.16	7.05 ± 0.69	7.06 ± 0.45	0.67 ± 0.38	4.98 ± 0.47	15.77 ± 0.72
OE-4	72.18 ± 4.14^*^	8.99 ± 0.51^*^	8.99 ± 0.26^*^	0.19 ± 0.65^*^	5.41 ± 0.36^*^	18.64 ± 0.08^*^
OE-6	72.77 ± 5.31^*^	8.64 ± 0.21^*^	8.31 ± 0.55^*^	0.26 ± 0.67^*^	5.48 ± 0.24^*^	17.54 ± 0.17^*^
Z44	69.15 ± 3.14	7.18 ± 0.54	7.19 ± 0.49	0.68 ± 0.39	4.95 ± 0.42	15.73 ± 0.68
OE-7	72.17 ± 4.05^*^	8.96 ± 0.58^*^	8.90 ± 0.30^*^	0.18 ± 0.64^*^	5.44 ± 0.34^*^	18.60 ± 0.06^*^
OE-9	71.77 ± 5.14^*^	8.26 ± 0.17^*^	8.20 ± 0.54^*^	0.29 ± 0.76^*^	5.39 ± 0.28^*^	17.97 ± 0.18^*^
Drought						
R15	42.05 ± 5.43	3.50 ± 0.69	2.48 ± 0.91	0.81 ± 0.24	4.05 ± 0.35	6.58 ± 0.15
OE-4	49.87 ± 4.73^**^	4.32 ± 1.24^*^	4.27 ± 1.13^**^	0.28 ± 0.19^*^	4.46 ± 0.28^*^	8.02 ± 0.69^*^
OE-6	48.38 ± 4.26^**^	3.94 ± 0.82^*^	3.90 ± 0.50^**^	0.31 ± 0.47^*^	4.19 ± 0.28^*^	7.95 ± 0.22^*^
Z44	41.99 ± 5.14	3.48 ± 0.62	2.42 ± 0.86	0.79 ± 0.92	4.06 ± 0.42	6.51 ± 0.19
OE-7	49.64 ± 4.76^**^	4.29 ± 1.21^*^	4.20 ± 1.15^**^	0.29 ± 0.18^*^	4.32 ± 0.24^*^	8.19 ± 0.67^*^
OE-9	48.72 ± 5.17^**^	4.02 ± 0.79^*^	3.99 ± 0.54^**^	0.34 ± 0.43^*^	4.28 ± 0.19^*^	8.01 ± 0.34^*^

n= 20 plants per line. Values are means ± SEs. ^*^, P< 0.05; ^**^, P< 0.01.

R15, Z44: Wild type receptor control R15 and Zhongmian 44; OE-4, -6, AtSTZ1-overexpressing transgenic cotton lines 4 and 6 generated using R15 as explants; OE-7, -9: AtSTZ1-overexpressing transgenic cotton lines 7 and 9 generated using Zhongmian 44 as explants.

### 3.2 *AtSTZ1* enhances drought tolerance in cotton

**Figure 2:**
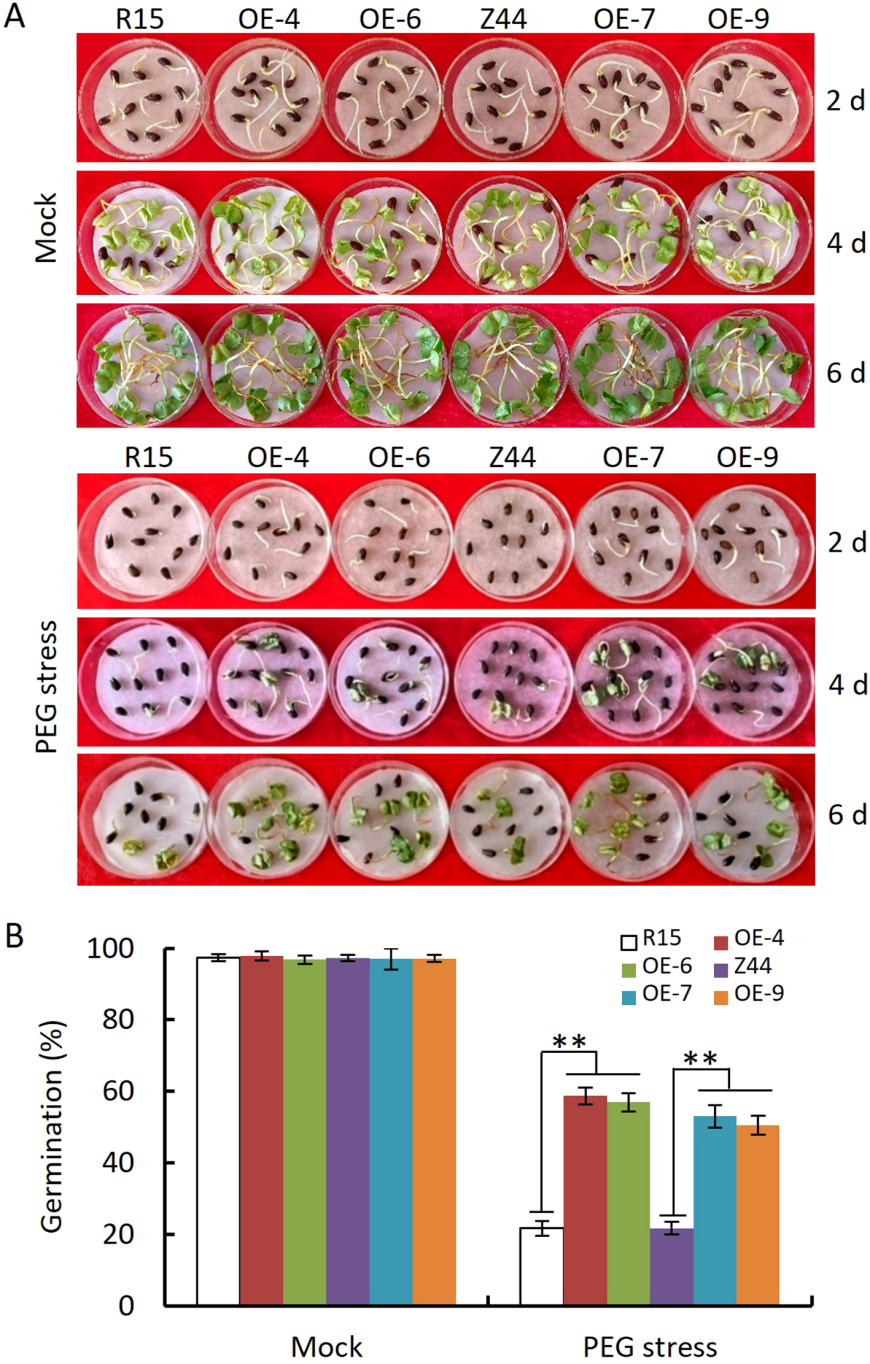
Assay of seed germination of independent T_3_ *AtSTZ1*-overexpressing transgenic cotton lines under PEG6000 treatment. **(A)** Growth status of wild-type and transgenic plants germinated in 10% PEG6000 for 2, 4, and 6 days. **(B)** Germination rate after 6 days of germination. The mean values and SEs (bars) of three independent experiments (n > 50 seeds per line) are shown. Independent *t-*tests revealed a very significant (**, P < 0.01) difference in the germination rate between the transgenic lines and the wild type under PEG6000 treatment. R15 and Z44, wild-type cotton (R15 and Zhongmian 44). OE-4, -6, *AtSTZ1-*overexpressing transgenic cotton lines 4 and 6 generated using R15 as explants; OE-7, -9: *AtSTZ1*-overexpressing transgenic cotton lines 7 and 9 generated using Zhongmian 44 as explants.

To evaluate whether *AtSTZ1*-overexpressing cotton plants exhibit enhanced drought tolerance, we conducted drought resistance tests during seed germination, seedling development, and the reproductive phase, respectively. Seeds from wild-type and *AtSTZ1*-overexpressing transgenic lines 4, 6, 7 and 9 were sown on 10% PEG6000-saturated filter papers (**Figure 2A**). **Figure 2B** shows the germination rates of the wild-type and *AtSTZ1* overexpression seeds in the condition containing 10% PEG6000. No significant variation was observed in the germination of *AtSTZ1*-overexpressing lines and wild type counterparts on sterilized water-saturated filter paper, with nearly complete germination in all seeds after 6 days (P ≥ 0.36, **Figures 2A, B**). Whereas, seeds from *AtSTZ1*-overexpressing plants germinated earlier and more rapidly than did from the wild-type controls (**Figures 2A, B**) under PEG6000 treatment. After 6 days, approximately 50-60% of the transgenic seeds had germinated, compared to only about 20% of the wild-type seeds (P ≤ 8.11E-10, **Figure 2B**). The above findings suggest that the *AtSTZ1* overexpressing transgenic cotton significantly enhanced drought tolerance during seed germination.

**Figure 3:**
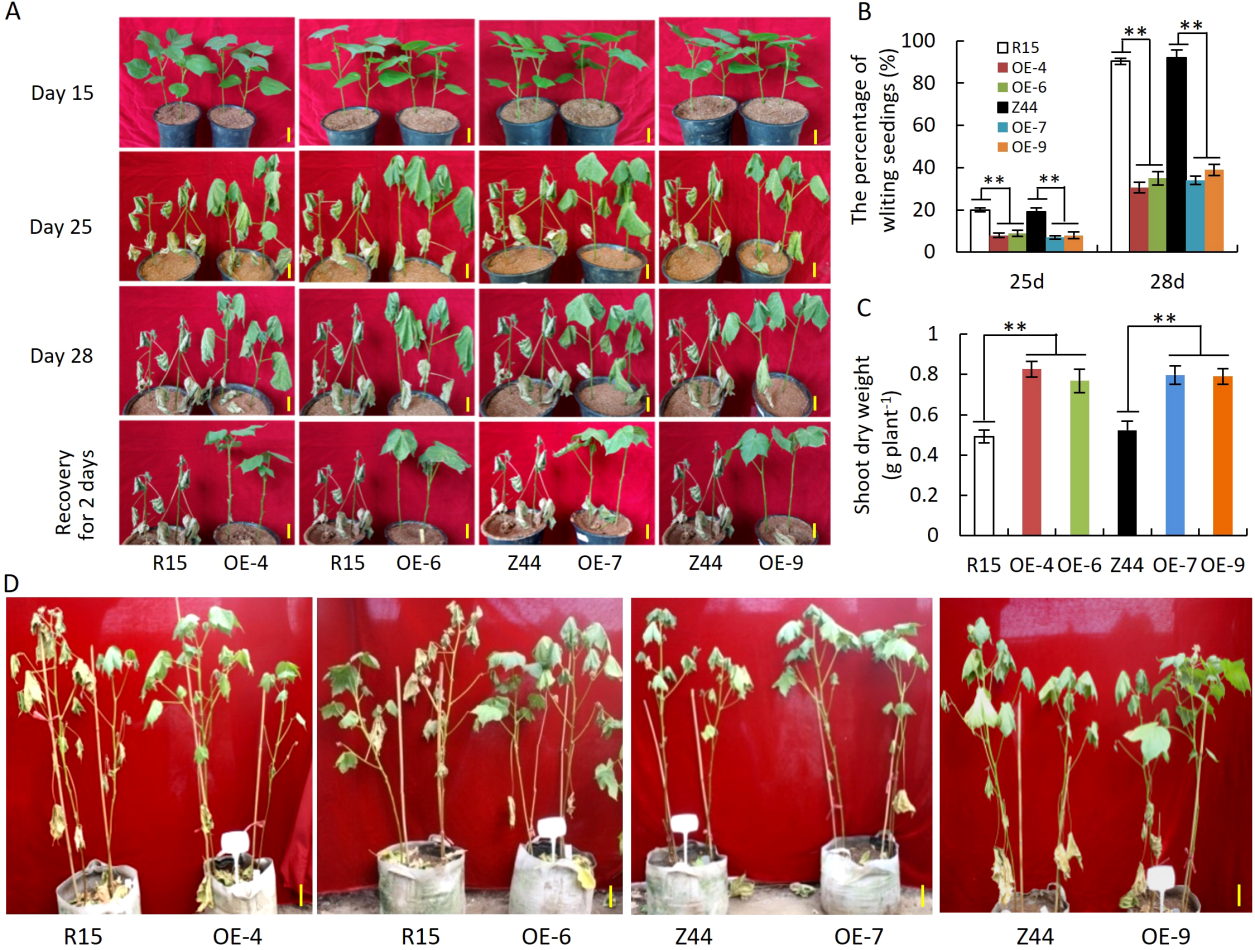
Significantly enhanced drought tolerance of *AtSTZ1* transgenic cotton seedlings in the greenhouse. **(A)** Growth of *AtSTZ1* transgenic cotton and wild-type cotton seedlings of the T_3_ generation after drought treatment for 15 days, 25 days, or 28 days and rehydration for 2 days in a greenhouse; **(B)** Wilting rate of transgenic cotton and wild-type seedlings after drought treatment for 25 days and 28 days; **(C)** Dry weight of transgenic cotton and wild-type seedlings after drought treatment for 28 days. The values are the means ± SEs of 60 plants (**, P < 0.01). **(D)** Phenotypes of *AtSTZ1*-overexpressing transgenic cotton plants at the reproductive stage in a greenhouse under drought stress. Plants were grown normally in planting bags for 35 days, and watering was then withheld for 40 days. Scale bars, 15 cm. R15, Z44: wild-type receptor control R15 and Zhongmian 44; OE-4, -6, *AtSTZ1-*overexpressing transgenic cotton lines 4 and 6 generated using R15 as explants; OE-7, -9: *AtSTZ1*-overexpressing transgenic cotton lines 7 and 9 generated using Zhongmian 44 as explants.

To assess the effect of *AtSTZ1* overexpression on drought tolerance, transgenic cotton plants with an overexpression of *AtSTZ1* were cultivated in a greenhouse under drought conditions. For evaluating drought resistance during the early seedling stage, seeds from four homozygous T_3_ generation lines (-4, -6, -7 and -9) of *AtSTZ1-*overexpressing transgenic cotton were planted in nutrition pots, and watering ceased when the cotton plants developed two cotyledons. As depicted in **Figure 3A**, no discernible difference was observed between the transgenic cotton lines and the wild-type plants during seedling development on the 15th day without watering. However, as drought stress persisted on days 25 to 28, cotyledons of the wild-type control were entirely shed, whereas *AtSTZ1* transgenic plants exhibited a significant delay in leaf-wilting phenotypes and reduced wilting percentages in contrast to those of wild type plants. Specifically, over 90% of the transgenic plants remained healthy (**Figures 3A, B**), and the wilting rate of the transgenic lines was only 7-8% (P ≤ 9.15E-5) after 25 days of water deprivation, while the wilting rate of the wild type controls was 20%. Following drought treatment for 28 days, the wilting rate (30-39%, P ≤ 8.74E-7) of the transgenic plants was notably lower than the wild-type (over 90%). After 28 days of drought stress and a subsequent two-day recovery period, the leaves of most of the transgenic plants returned to normal and still grew healthily, whereas almost all of the wild type plants fully wilted and even died (**Figures 3A, B**). In addition, we observed a significant increase (0.56-0.68 fold) in the dry biomass of the *AtSTZ1* overexpressing transgenic lines under drought stress in comparison to the wild-type counterparts (P ≤ 3.83E-12, **Figure 3C**).

To assess drought resistance during the reproductive phase, thirty-five days old greenhouse-grown plants with well-watering went through a 40-day period without watering. As shown in **Figure 3D**, the wild-type control exhibited a pronounced drought-stress phenotype, with leaves almost completely wilted. In contrast, all four transgenic lines maintained a relatively normal state of growth. These findings indicate that *AtSTZ1* can dramatically improve the drought tolerance of cotton plants in different growth and development periods. On the whole, our results indicate that overexpression of *AtSTZ1* improves drought resistance in cotton.

### 3.3 *AtSTZ1* improves enlarged root system in cotton

**Figure 4:**
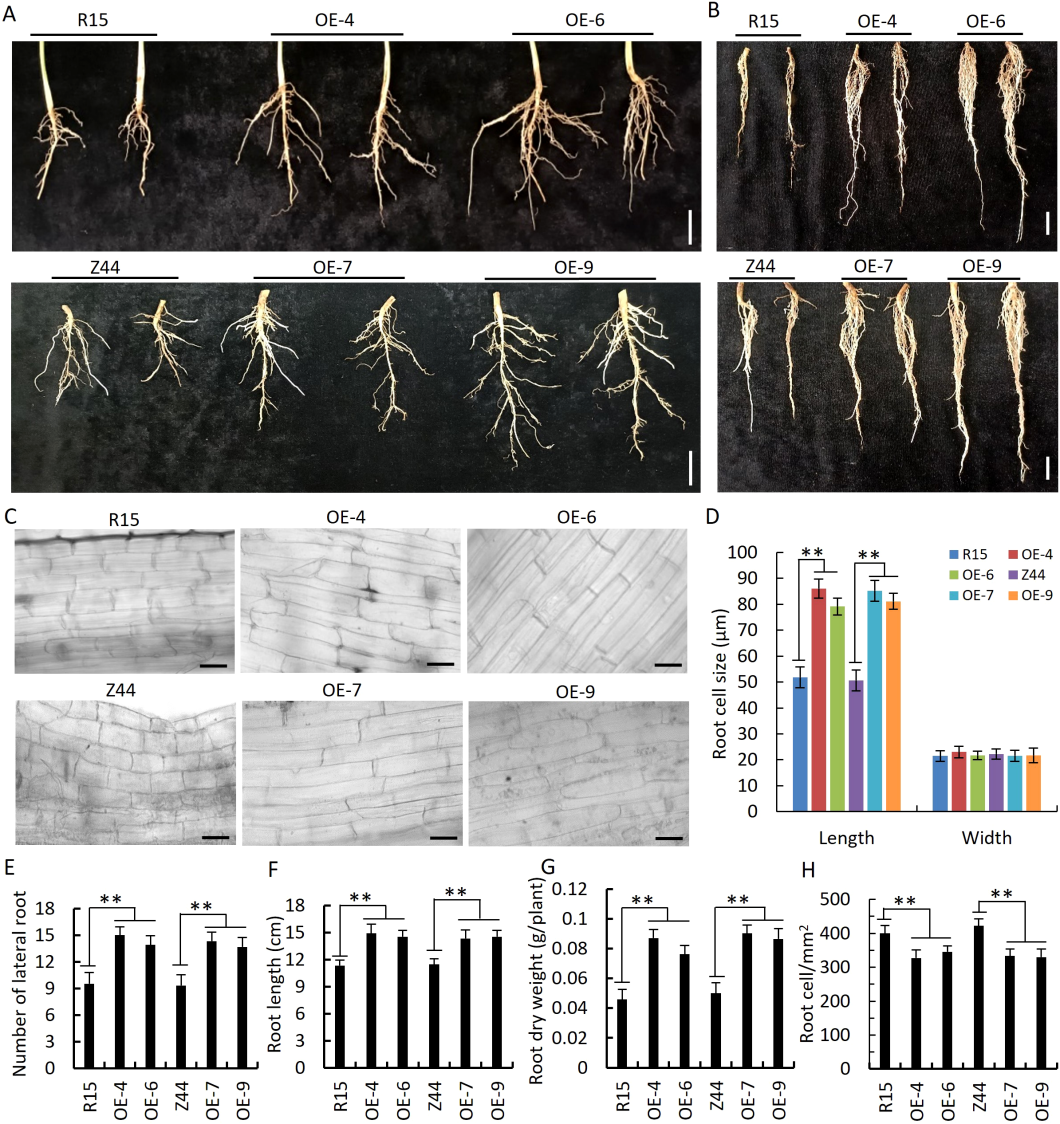
Enlarged root system of *AtSTZ1* transgenic cotton plants. **(A, B)** Roots of *AtSTZ1*-overexpressing transgenic and wild-type cotton plants grown under normal conditions for 15 days **(A)** and treated with drought for 25 days **(B)**. Scale bars, 3 cm. **(C)** The number of lateral roots on 15-day-old wild-type and *AtSTZ1* transgenic cotton plants. **(D)** Micrographs of root cells from wild-type and *AtSTZ1* transgenic plants. Scale bars, 50 µm. **(E, F)** Primary root length and root dry weight of plants subjected to drought for 25 days. **(G, H)** Mean number and size of root cells from wild-type and *AtSTZ1* transgenic plants. R15 and Z44, wild-type cotton (R15 and Zhongmian 44). OE-4, -6, *AtSTZ1-*overexpressing transgenic cotton lines 4 and 6 generated using R15 as explants; OE-7, -9: *AtSTZ1*-overexpressing transgenic cotton lines 7 and 9 generated using Zhongmian 44 as explants. The values are the means ± SEs of three replicates (**, P < 0.01).

We observed that four AtSTZ1-overexpressing transgenic cotton lines (-4, -6, 7, and -9) exhibited stronger growth than the wild-type controls in the field in Yuncheng, Shanxi Province, China (**Figures 1C, F**; **Supplementary Figure S2**). The *AtSTZ1*-overexpressing transgenic cotton plants not only produced significantly larger bolls (**Figures 1C, F**; **Supplementary Figure S2**), but also developed an expanded root system (**Figures 1C, F**; **Figures 4A, B**, the roots were cut from AtSTZ1-overexpressing transgenic cotton plants treated with drought for 15 days and 25 days, respectively) characterized by increased length of primary roots (**Figures 4B, E**; P ≤ 5.81E-8) and increased numbers of lateral roots (**Figure 4C**, P ≤ 2.8E-10), significantly augment of root dry biomass (**Figure 4F**, P ≤ 1.61E-7), and an increase of roots cell size in length (**Figures 4D, H**; P ≤ 4.92E-51). Therefore, the average number of root cells per apparent area in transgenic lines 4 and 6 was obviously less than the wild type control (**Figure 4G**, P ≤ 2.25E-11). This enhanced root system could potentially benefit cotton plant growth and drought tolerance.

### 3.4 *AtSTZ1*-overexpressing cotton exhibits decreased stomatal density and transpiration rate with increased photosynthetic rate and water-use efficiency in leaves

**Figure 5:**
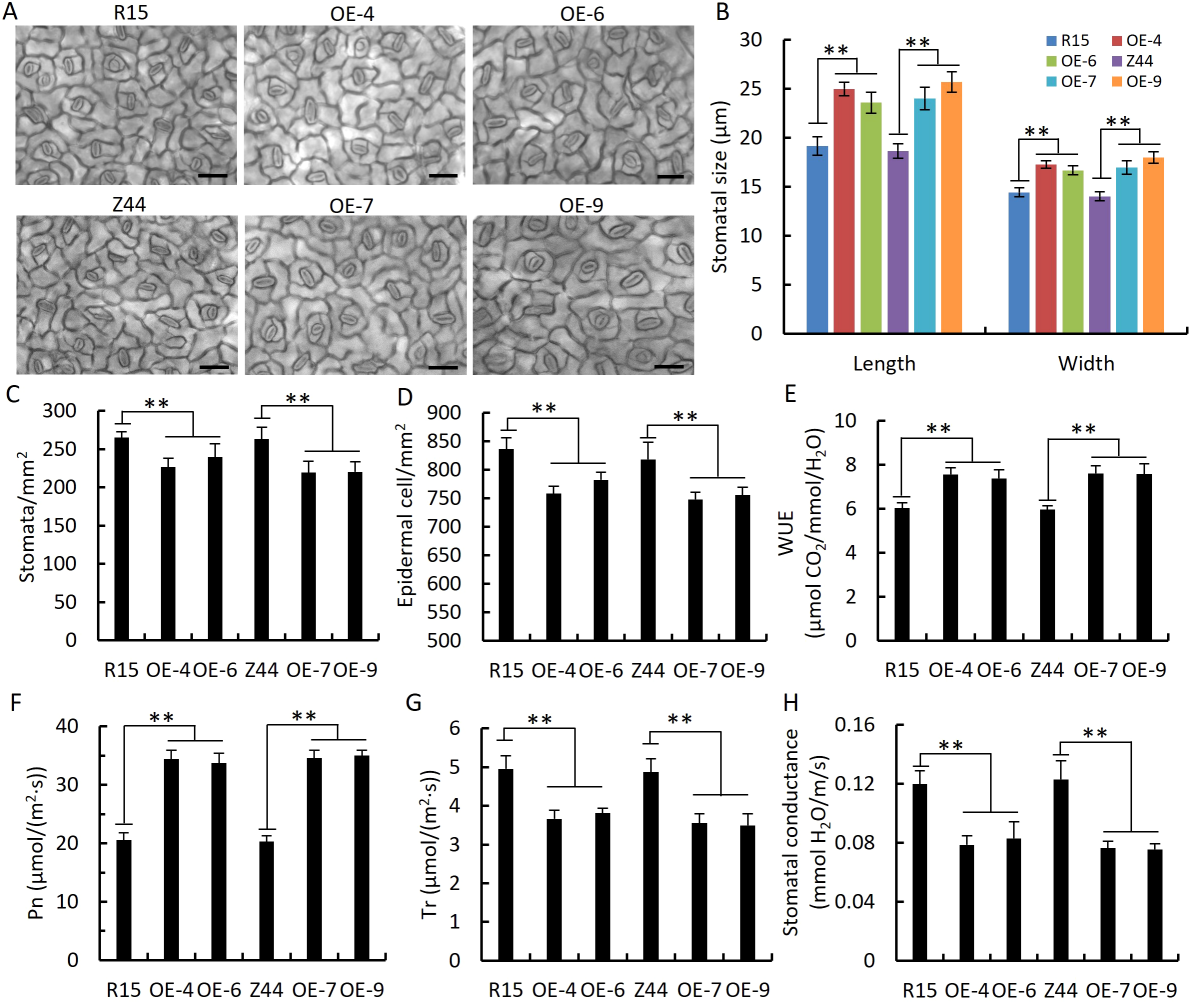
Decreased stomatal density, stomatal conductance, and transpiration rate; increased stomatal and epidermal cell size; and increased water use efficiency (WUE) and photosynthetic rate in *AtSTZ1*-overexpressing transgenic cotton. **(A)** Micrograph of the adaxial epidermal cells of wild-type and *AtSTZ1* transgenic cotton plants. Bar = 50 μm. **(B)** Stomatal size of wild-type and *AtSTZ1* transgenic plants. Stomata were counted and measured by microscopy. The values are the means ± SEs of 50 stomata. **(C, D)** Stomatal density and epidermal cell number of wild-type and transgenic plants (n = 50 images per line). **(E, H)** WUE **(E)** and stomatal conductance **(H)** of wild-type and transgenic plants. **(F-G)** Photosynthetic rate (Pn, **F**) and transpiration rate (Tr, **G**) of wild-type and transgenic plants (n=10 fully expanded leaves per line). R15 and Z44, wild-type cotton (R15 and Zhongmian 44). OE-4, -6, *AtSTZ1-*overexpressing transgenic cotton lines 4 and 6 generated using R15 as explants; OE-7, -9: *AtSTZ1*-overexpressing transgenic cotton lines 7 and 9 generated using Zhongmian 44 as explants. The mean value was ± SEs (**, P < 0.01).

We measured stomatal size and number in the leaves of *AtSTZ1*-overexpressing transgenic plants and the wild type controls. Both the length (P ≤ 1.66E-35) and width (P ≤ 3.26E-26) of the stomatal guard cells were larger in two transgenic cotton lines than the wild-type control (**Figures 5A, B**). The average stomatal densities of the transgenic lines 4, 6, 7 and 9 were respectively 26.04%, 22.26%, 28.45% and 27.13% less than in the wild type counterparts, respectively (**Figures 5A, C**; P ≤ 1.91E-16). The observation that epidermal cells in the transgenic lines overexpressing *AtSTZ1* was larger compared to wild type (P ≤ 2.49E-9), which resulted in a notable decrease in stomatal density of transgenic lines (**Figure 5D**). Consequently, the *AtSTZ1*-overexpressing plants exhibited greater water-use efficiency (WUE, **Figure 5E**, P ≤ 4.72E-6), greater photosynthetic rates (**Figure 5F**, P ≤ 4.17E-9), lower stomatal conductance (**Figure 5G**, P ≤ 2.36E-5), and lower transpiration rates (**Figure 5H**, P ≤ 3.98E-5) than did the wild-type controls. Accordingly, those data revealed that the increase in WUE of the transgenic cotton lines was attributable to a decrease in stomatal density and conductance.

### 3.5 *AtSTZ1* transgenic plants show improved stress tolerance parameters

**Figure 6:**
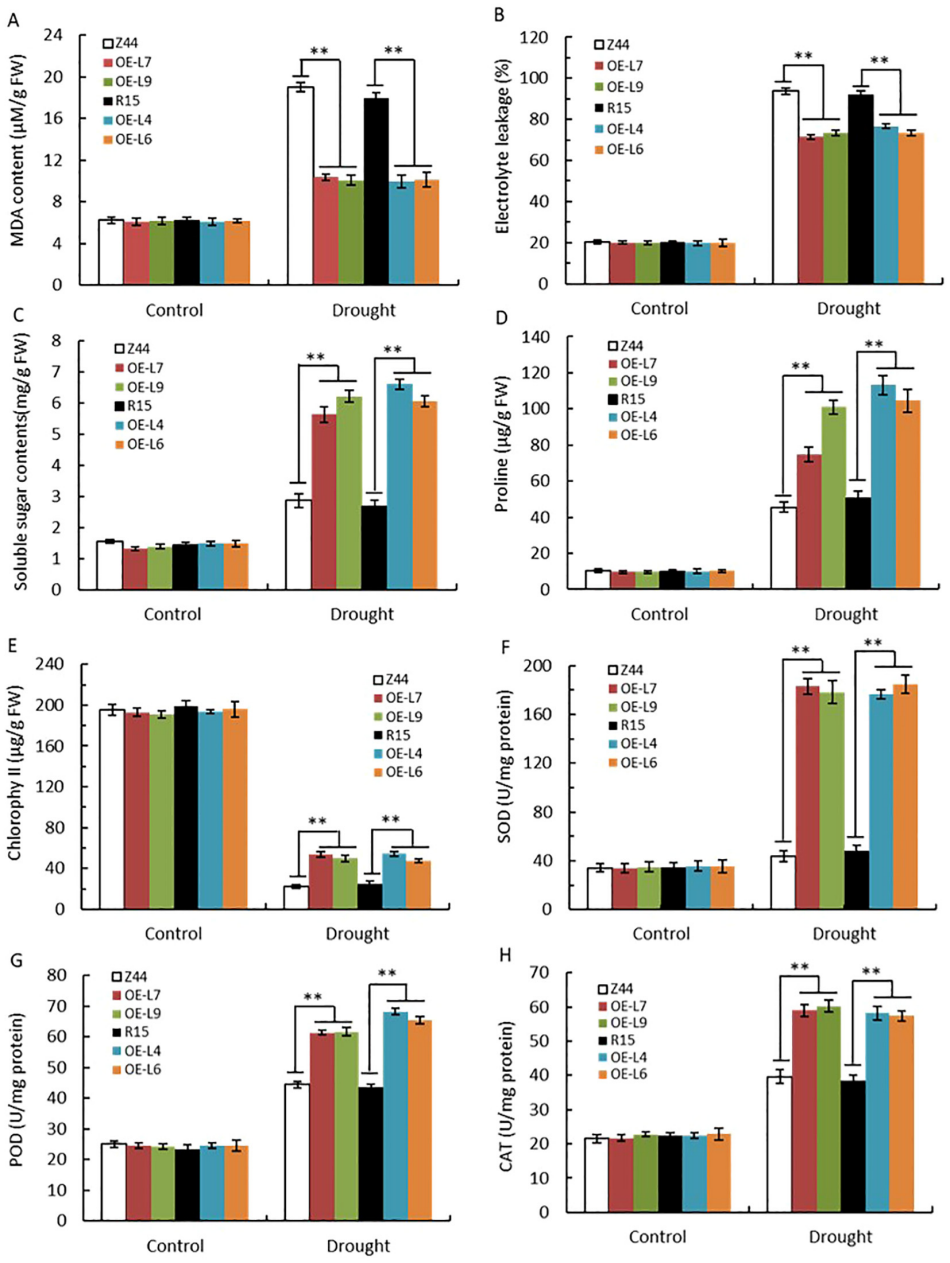
Determination and analysis of physiological indices of *AtSTZ1* transgenic cotton after drought treatment. The malondialdehyde (MDA) content **(A)**, electrolyte leakage **(B)**, soluble sugar **(C)**, proline content **(D)**, chlorophyll content **(E)**, and SOD **(F)**, POD **(G)** and CAT **(H)** activities of the transgenic cotton plants and wild-type plants were measured. The values are the means ± SEs of three replicates (**, P < 0.01). Plants (approximately 20 plants per line) were treated for 25 days with or without irrigation. Mean values and SEs (bars) from three independent experiments (n = 20 fully expanded leaves per line). Independent *t-*tests for comparison of mean values showed significant differences between wild-type and transgenic plants (**, P < 0.01). R15, Z44: wild-type receptor control R15 and Zhongmian 44; OE-4, -6, *AtSTZ1-*overexpressing transgenic cotton lines 4 and 6 generated using R15 as explants; OE-7, -9: *AtSTZ1*-overexpressing transgenic cotton lines 7 and 9 generated using Zhongmian 44 as explants.

We compared several physiological parameters between *AtSTZ1* transgenic lines and the wild type plants following drought treatment. Under normal well-watered conditions, malondialdehyde (MDA), proline, chlorophyll, and soluble sugar contents; electrolyte leakage; and peroxidase (POD), catalase (CAT), and superoxide dismutase (SOD) activities were similar in both the transgenic lines and the wild-type control (**Figure 6**). However, following drought stress, the MDA content (**Figure 6A**, P ≤ 1.83E-7) and electrolyte leakage (**Figure 6B**, P ≤ 1.97E-5) in the wild-type cotton plants were greater than those in the transgenic lines, suggesting that there was less oxidative damage in the transgenic cotton plants than in the wild-type control plants. Moreover, the contents of soluble sugars (**Figure 6C**, P ≤ 0.0002), proline (**Figure 6D**, P ≤ 0.0001) and chlorophyll (**Figure 6E**, P ≤ 2.68E-6) in the *AtSTZ1*-overexpressing cotton lines increased significantly relative to the wild-type control after drought treatment. Notably, the activities of reactive oxygen species (ROS)-scavenging enzymes, such as SOD (**Figure 6F**, P ≤ 1.51E-5), POD (**Figure 6G**, P ≤ 2.08E-5) and CAT (**Figure 6H**, P ≤ 4.26E-6), were markedly greater in the transgenic lines compared to those of in the wild type plants following drought treatment. Hence, our data display that the transgenic cotton lines overexpressing *AtSTZ1* exhibited an enhanced drought resistance in comparison to the wild-type plants, likely because it better protects against oxidative damage during drought stress.

### 3.6 *AtSTZ1* influences the expressions of multiple transcription factors and drought-responsive genes

**Figure 7:**
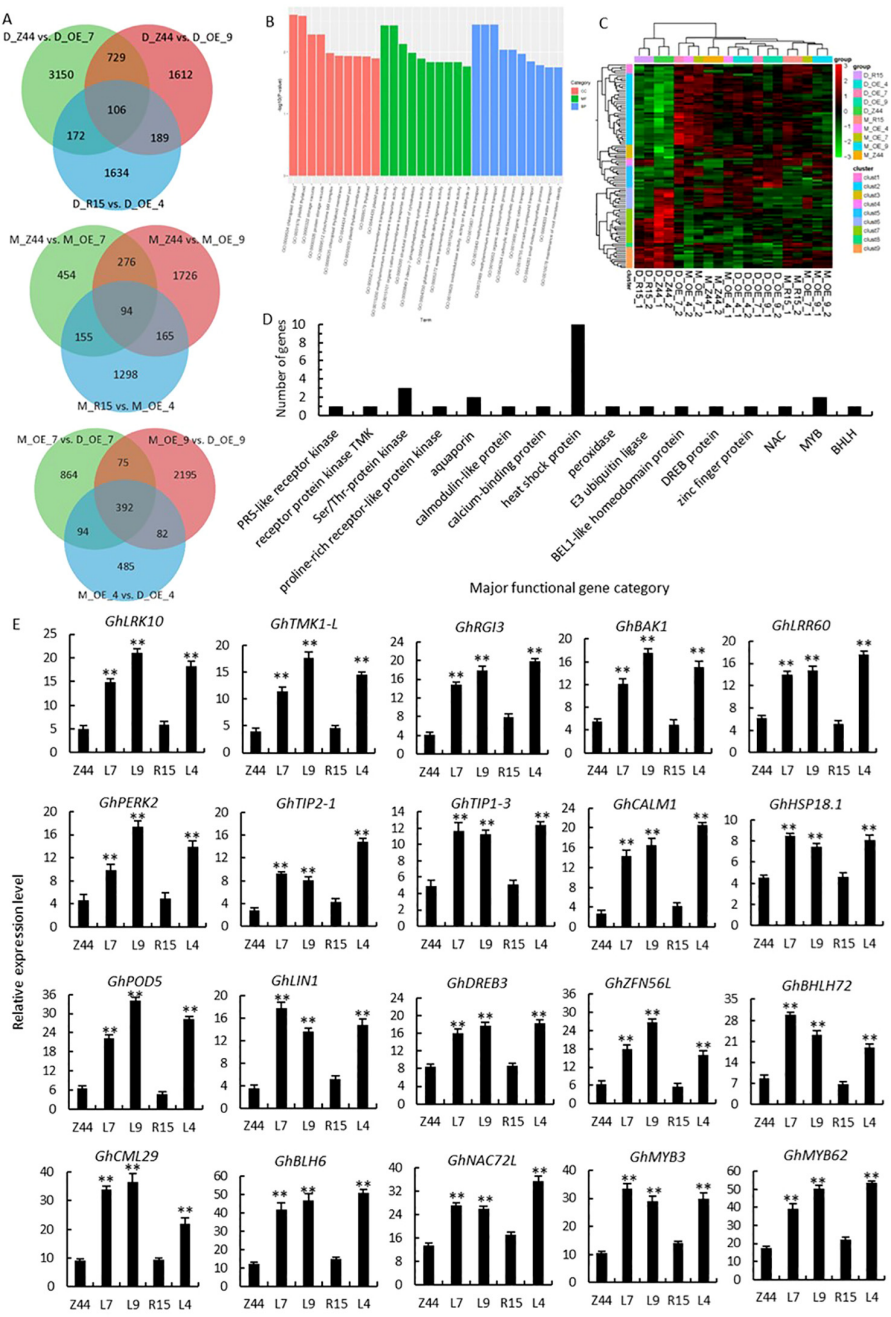
Major classes of functional genes differentially upregulated in *AtSTZ1*-overexpressing transgenic cotton under drought stress. **(A)** Venn diagram of unigenes identified as differentially expressed between the wild-type and transgenic lines (control vs drought). **(B)** Gene Ontology (GO) functional classification of 106 differentially expressed genes (DEGs). BP, biological process; MF, molecular function; CC, cellular component. **(C)** Heatmaps showing the transcript levels of 106 DEGs. The columns and rows in the heatmaps represent the samples and DEGs, respectively. Red indicates genes with high expression levels, and green indicates genes with low expression levels. **(D)** Major classes of the upregulated functional genes in the transgenic lines according to the RNA-seq data. **(E)** Quantitative RT-PCR analysis of the 20 selected major genes. *GhLRK10* (PR5-like receptor kinase, GH_A01G0439), *GhTMK1-L* (receptor protein kinase TMK1-like, GH_A01G2189), *GhRGI3*, *GhBAK1* and *GhLRR60* (LRR receptor-like serine/threonine-protein kinase, GH_A04G0041, GH_A12G2843 and GH_A02G1960), *GhPERK2* (proline-rich receptor protein kinase, GH_D02G0727), *GhTIP2-1*, *GhTIP1-3* (aquaporin, GH_D03G0641 and GH_D13G2562), *GhCML1* (calmodulin-like protein 1, GH_D12G2948), *GhCML29* (calcium-binding protein CML29, GH_A05G2319), *HSP18.1* (18.1 kDa class I heat shock protein, GH_A09G1779), *GhPOD5* (peroxidase 5, GH_D12G2674), *GhLIN1* (E3 ubiquitin-protein ligase, GH_D09G2516), *GhDREB3L* (dehydration-responsive element-binding protein 3-like, GH_D06G1018). The values are the means ± SDs of three replicates. Z44 and R15, wild-type receptor controls Zhongmian 44 and R15; OE-4 (L4), *AtSTZ1-*overexpressing transgenic cotton lines 4 generated using R15 as explants; OE-7 (L7), and OE-9 (L9), *AtSTZ1*-overexpressing transgenic cotton lines 7 and 9 generated using Zhongmian 44 as explants. M, mock, under normal growth conditions; D, under drought treatment. Student’s *t-*tests revealed significant differences (**, P < 0.01) among the transcript levels.

In order to deeper explore the regulatory function of *AtSTZ1* in cotton in responding to drought stress, we performed transcriptomic analysis to identify differentially expressed genes (DEGs) under both irrigated and drought conditions. A Venn diagram (**Figure 7A**) displays the DEGs in transgenic lines 4, 7 and 9 and in the wild-type plants (R15 and Zhongmian 44) (control vs drought). A total of 5860 DEGs (including 2906 downregulated and 2954 upregulated genes) and 5805 DEGs (comprising 2824 downregulated and 2981 upregulated genes) were identified in the wild-type controls (Zhongmian 44 and R15) following drought treatment, respectively (**Supplementary Table S2**). In *AtSTZ1*-overexpressing lines 4, 7 and 9, 392 DEGs, including 188 downregulated genes and 204 upregulated genes, were identified under drought stress (**Supplementary Table S2**; **Supplementary Appendix S1**; **Figure 7A**). 94 DEGs (comprising 42 downregulated and 52 upregulated genes) were identified in three *AtSTZ1*-overexpressing lines and the wild type plants following non-stressful growth environments. In contrast to those in the wild type controls, 106 DEGs (including 56 downregulated and 60 upregulated genes) were identified in those three *AtSTZ1*-overexpressing lines under drought treatment (**Supplementary Appendixes S1**-**2**; **Supplementary Table S2**; **Figure 7A**). The differentially regulated transcripts of these 106 DEGs are engaged in a variety of biological processes, cellular compartments, and molecular functions according to the functional classification via GO enrichment (**Figure 7B**). The expression levels of these 106 DEGs in *AtSTZ1*-overexpressing lines 4, 7 and 9 and the wild-type controls (Zhongmian 44 and R15) following non-irrigated conditions are depicted in heatmaps (**Figure 7C**).

Among the 106 DEGs, 60 were upregulated (**Figure 7D**; **Supplementary Table S3**), encompassing major functional gene classes. These included an E3 ubiquitin-protein ligase (GH_D09G2516, associated with stomatal conductance) (Tong et al., 2021), a calmodulin protein (GH_D12G2948), a calcium-binding protein (GH_A05G2319), and a peroxidase (GH_D12G2674, associated with root antioxidation) (Luo et al., 2016). Two aquaporins (GH_D03G0641 and GH_D13G2562) related to the regulation of stomatal response and root growth have been recognized (Kapilan et al., 2018; Guo et al., 2022). The expression of six kinases, including a PR5-like receptor kinase (GH_A01G0439, associated with stomatal conductance) (Baek et al., 2019), a proline-rich protein kinase (GH_D02G0727), a receptor protein kinase (GH_A01G2189, linked to regulating root hair growth) (Wei and Li, 2018), and three Ser/Thr protein kinases (GH_A04G0041, GH_A12G2843 and GH_A02G1960), were also upregulated. Seven transcription factors, comprising an AP2/ERF dehydration-responsive element-binding protein (GH_D06G1018, associated with root development) (Lee et al., 2016; El-Esawi and Alayafi, 2019), a zinc finger protein (GH_A07G1346, linked to regulating the function of stomata) (Han et al., 2021), a BLH/BELL transcription factor (GH_D13G0313, regulating secondary cell wall biosynthesis) (Ma et al., 2019), a bHLH (GH_D03G1091, associated with stomatal development) (Yang et al., 2023), a NAC (GH_A01G0644, linked to root development) (Liu et al., 2014), and two MYBs (GH_D08G0405 and GH_D12G2894, controlling stomatal size and opening) (Butt et al., 2017; Chen et al., 2015) were identified. Additionally, 10 heat shock proteins (GH_A09G1779, GH_A02G0345, GH_A07G0271, GH_A09G2360, GH_D08G1971, etc.) linked to ROS scavenging (Hou et al., 2018; Zhang and Huang, 2020), were dramatically upregulated in *AtSTZ1-*overexpressing lines following drought conditions. Our RNA-seq results are in alignment with the observed phenotypic traits exhibited by the transgenic lines.

To verify the accuracy of drought-response relative gene expression patterns of the DEGs, the expression levels of 20 representative upregulated DEGs from the *AtSTZ1*-overexpressing plants were determinated by qRT-PCR (**Figure 7E**). The qRT-PCR validation of the transcriptional levels of the drought-related genes screened exhibited congruence with the RNA-seq transcript abundance data, demonstrating the accuracy and reliability of the transcriptomic data.

## 4 Discussion

The major objective of crop breeding is to increase quality and yield. However, drought significantly impedes crop growth, development and total yield. Thus, the cultivation of crop varieties possessing enhanced drought resilience is essential for boosting overall crop productivity. Numerous genes have been isolated and identified as potential candidates for genetic modification to combat drought stress. These genes include genes encoding metabolites or penetrant protectants, genes related to ion homeostasis, hormone genes associated with signal transduction and genes encoding transcription factors (Yang et al., 2010; Umezawa et al., 2006; Liu et al., 2014). However, the overwhelming majority of genetic improvements have been detected in *Arabidopsis thaliana*, with only minor genes transformed into crops (Peleg et al., 2011). Genetic engineering technology has emerged as an efficient and fast approach in crop breeding, nevertheless, the ectopic expression of most stress-responsive genes usually leads to aberrant growth, development and yield losses (Priyanka et al., 2009; Nakashima et al., 2007). Encouragingly, *AtHDG11* overexpressing transgenic cotton has demonstrated significant feasibility in increasing agricultural yield in the case of drought or water scarcity (Yu et al., 2016). The function of *AtSTZ1/ZAT10* involving in regulating drought stress response in *Arabidopsis* has produced diametrically opposite results. Sakamoto et al. (2004) demonstrated that the overexpression of *AtSTZ1* can notably improve drought resilience of *Arabidopsis* plants. Conversely, Woo et al. (2008) reported that the drought tolerance of transgenic *Arabidopsis* expressing *AtSTZ1* was obviously enhanced via RNA interference. In our study, *AtSTZ1-*overexpressing transgenic cotton plants yielded similar results to those in which *AtHDG11* was overexpressed in cotton, with no transparent abnormal or negative growth or development observed. In contrast, *AtSTZ1*-overexpressing transgenic cotton plants exhibited an enlarged root system (**Figures 1F**, **4**), increased stomata and epidermal cells in leaves, and decreased stomatal density (**Figure 5**). Importantly, in field trials, *AtSTZ1* overexpression increased fiber yield, enhanced agronomic properties, and improved drought tolerance (**Figure 1**, **Table 1**), both in conditions of sufficient water and natural drought. In contrast to the wild type plants, transgenic cotton lines overexpressing *AtSTZ1* in the field displayed a clear growth advantage, as indicated by significantly greater total cotton yield, boll number, fruit branch number, and plant height following sufficient water and natural drought stress (**Table 1**). Fiber yields from *AtSTZ1-*overexpressing transgenic lines 4, 6, 7 and 9 increased by 21.88%, 20.82%, 25.81% and 23.04% after drought stress and by 18.20%, 11.22%, 18.25% and 14.24% under normal irrigation conditions, respectively (**Table 1**). The transgenic lines exhibited a more pronounced increase in relative cotton fiber yield during drought stress compared to normal well-watered conditions (**Table 1**). Consequently, our data pointed towards the greater benefits of *AtSTZ1* overexpression in cotton for improved plant growth following drought condition, and our research identified a hopeful candidate gene, *AtSTZ1*, for overcoming the extreme environment faced by cotton production. As a potential candidate gene, *AtSTZ1* can be served to enhance the drought resilience of crops in certain regions where the output of agriculture is constrained by the supply of water, and the excellent agronomic performance of *AtSTZ1*-overexpressing transgenic cotton plants contributes to some crops in the context of water scarcity.

Prior studies have attributed the increase in drought resistance associated with *AtHDG11* to the development of the stomatal density and root system, increased WUE and photosynthesis, and improved tolerance to oxidative stress (Yu et al., 2016). Likewise, our research shows that the enhanced drought resistance of *AtSTZ1*-overexpressing transgenic cotton plants is due to morphological and physiological changes. Firstly, the expanded root system of transgenic plants maximizes nutrient and water absorption in drought-stressed conditions. One potential explanation for the root changes in transgenic plants is phosphorylation regulation of the receptor protein kinase TMK1-like (TMK1-L) (GH_A01G2189) (**Figure 7**), a critical regulatory factor of root development in *Arabidopsis thaliana* (Wei and Li, 2018). Furthermore, the reductions in transpiration rate, stomatal density and conductivity (**Figure 5**) in the transgenic cotton plants likely contributed to an enhanced WUE (**Figure 5E**) and improved water retention capacity. Additionally, the decreased electrolyte leakage and MDA content of *AtSTZ1*-overexpressing transgenic cotton lines, coupled with increased soluble sugar, free proline and chlorophyll contents; and CAT, POD and SOD activities, better protected the plants from oxidation and osmotic damage (**Figure 6**).

Previous reports have demonstrated that *AtZAT12* can upregulate the expression of *APX1*, *ZAT7* and *WRKY25* (Wang et al., 2020). *ZAT10/AtSTZ1* is controlled by MPK3 and MPK6 phosphorylation and participates in multiple abiotic stress responses (Sakamoto et al., 2004; Nguyen et al., 2016). Tian et al. (2024) reported that the zinc finger protein DHHC09 modulates kinase STRK1 through S-acylation, regulating H_2_O_2_ levels and improving salt resistance in rice. In the present study, transcriptome data unveiled that the transcriptional levels of several kinases in *AtSTZ1*-overexpressing transgenic cotton lines increased following drought treatment. Consequently, we hypothesize that *AtSTZ1* is regulated at the post-translational level. Besides, the upregulated expression of the stress-related genes *GhCALM1*, *GhCML29*, *GhPOD5*, and *GhHSPs* in *AtSTZ1*-overexpressing transgenic cotton lines suggested that under drought stress, AtSTZ1 functions as a positive modulator of genes responding to abiotic stress. Li et al. (2023) confirmed that the expression of *MhZAT10* was directly regulated and activated by *MhDREB2A* responding to drought stress. Furthermore, the overexpression of *MhDREB2A* and *MhZAT10* improved the resistance of plants to cold and drought. Subsequently, *MhMYB124* and *MhMYB88* were identified as downstream target genes regulated by *MhZAT10*. In this study, our transcriptome-seq data and qRT-PCR assays revealed the upregulation of diverse transcription factor (comprising GhDREB3, GhBLH6, GhZFN56L, GhNAC72L, GhMYB3, GhMYB62 and BHLH72) in the transgenic cotton plants, though the precise role and positioning of *AtSTZ1* in the transcriptional regulatory network is still unclear. Therefore, we speculate that the expression of *AtSTZ1* could potentially be directly controlled and activated by GhDREB3 responding to drought stress. Moreover, *GhMYB3* and *GhMYB62* may be downstream regulatory target genes of *AtSTZ1* that involving in drought signal response. Additionally, the transcription level S*NAC1* gene from rice significantly increased in guard cells under drought stress, and the overexpression of *SNAC1* in cotton improves drought resistance via promoting root growth and decreasing the transpiration rate (Liu et al., 2014). Similarly, *AtSTZ1*-overexpressing transgenic cotton lines exhibited improved drought resistance, and had an expanding root system, and a reduced transpiration rate in comparison to the wild-type counterparts. Thus, we speculate that upregulated expression of *AtSTZ1*, controlled by *GhNAC72L*, improves the development of root system and decreases the transpiration rate, thereby enhancing the drought resistance of cotton.

In conclusion, our researches demonstrated that the overexpression of *AtSTZ1* in cotton strengthened its drought resistance. *AtSTZ1* transgenic cotton lines exhibit several traits linked to drought resistance, including a better-developed root system; enlarged stomatal size; enhancive photosynthetic rate and WUE; elevated CAT, POD and SOD activities; increased proline and chlorophyll contents; and reduced MDA levels, transpiration rate and stomatal density. Transgenic cotton overexpressing *AtSTZ1* exhibited enhanced drought tolerance, better agronomy characteristics, and increased fiber production in the field environment. Additionally, underlying transcription factors and drought-responsive genes related to *AtSTZ1* were identified. Regardless of the mechanism through which *AtSTZ1* responds to drought, the satisfying agronomic traits obtained in *AtSTZ1*-overexpressing transgenic cotton lines will benefit crops under water-limited conditions. Moreover, the overexpression of *AtSTZ1* in *Arabidopsis*, tobacco and cotton significantly improved plant drought resistance (Mittler et al., 2006; Sakamoto et al., 2004). Overexpression of *MhZAT10* in cold-sensitive cultivar ‘G935’ promoted overwintering sprouting, while the overexpression of *MhZAT10* and *MhDREB2A* enhanced plant resistance to cold and drought (Li et al., 2023). To sum up, these studies suggest that the drought tolerance and other phenotypes conferred by the dicot *ZAT10/STZ1* are likely conserved in other crop species. This finding implies that altering the expression pattern of *AtSTZ1* may be a natural evolutionary strategy for drought tolerance, and this gene holds great potential for use in crop genetic improvement.

## 5 Conclusion

Overexpression of *AtSTZ1* in cotton produced increased agronomic performance and fiber yield in the field, as well as improved drought resistance. The transgenic cotton plants displayed a variety of traits associated with drought tolerance, comprising a larger and more extensive root system, enlarged stomatal aperture, decreased stomatal density, increased WUE, heightened photosynthetic rate, and increased CAT, POD and SOD activities, along with increased chlorophyll, soluble sugar, and proline contents. Additionally, *AtSTZ1* upregulated the expression of abiotic stress-related genes in cotton responding to drought.

## Data Availability

The datasets presented in this study can be found in online repositories. The names of the repository/repositories and accession number(s) can be found below: NCBI(SAR)- PRJNA1171793; PRJNA1171421; PRJNA1171940. https://doi.org/10.6084/m9.figshare.27088918.

## Supplementary material

Supplementary Appendix S1 Differentially expressed genes identified in *AtSTZ1*-overexpressing transgenic lines (Control-vs-Drought).

Supplementary Appendix S2 106 differentially expressed genes of *AtSTZ1*-overexpressing transgenic cotton lines in comparison with the wild type following drought stress.
