# Supplementary material for: AtZAT10/STZ1 improves drought tolerance and increases fiber yield in cotton
Source: Front Plant Sci. 2024 Oct 21;15:1464828. doi: 10.3389/fpls.2024.1464828 (PMC11532130; doi:10.3389/fpls.2024.1464828)
Supplement: Supplementary Appendix S1 — Differentially expressed genes identified in AtSTZ1-overexpressing transgenic lines (Control-vs-Drought). [file DataSheet1.pdf]

| id | baseMean A | baseMean B | baseMean C | log2FoldChange | pval |
|---|---|---|---|---|---|
| GH_D12G2239 | 5302.969008 | 1.014883893 | 10604.92313 | 13.35113186 | 2.50763E-81 |
| GH_D06G1267 | 1119.968269 | 0.507441947 | 2239.429095 | 12.10760059 | 3.124E-59 |
| GH_D06G1266 | 176.0941791 | 0 | 352.1883581 | Inf | 2.78351E-30 |
| GH_A11G1929 | 145.3333594 | 4.273689011 | 286.3930298 | 6.066370641 | 6.46065E-21 |
| GH_A09G1680 | 85.63052938 | 0 | 171.2610588 | Inf | 2.10058E-20 |
| GH_A13G1414 | 79.23797093 | 0 | 158.4759419 | Inf | 2.05539E-19 |
| GH_D03G0833 | 69.39000171 | 0 | 138.7800034 | Inf | 8.20619E-18 |
| GH_A10G2376 | 440.0129705 | 61.88823253 | 818.1377084 | 3.724606672 | 7.38806E-16 |
| GH_D06G2079 | 771.2352535 | 137.9674007 | 1404.503106 | 3.347660486 | 1.17694E-14 |
| GH_D03G1716 | 453.1138046 | 79.40772864 | 826.8198805 | 3.380221743 | 1.70007E-14 |
| GH_A10G2379 | 132.2295189 | 13.39601816 | 251.0630197 | 4.2281734 | 1.67295E-13 |
| GH_A08G0271 | 76.54535586 | 6.230134671 | 146.8605771 | 4.559040015 | 1.7476E-11 |
| GH_D09G0167 | 51.29490606 | 0.941561766 | 101.6482504 | 6.754313929 | 2.3099E-11 |
| GH_D10G2470 | 207.0529395 | 42.78696047 | 371.3189186 | 3.117415722 | 2.42639E-10 |
| GH_A11G0919 | 218.3691671 | 52.29915203 | 384.4391821 | 2.877895923 | 1.19555E-09 |
| GH_A09G0886 | 36.7652463 | 1.014883893 | 72.5156087 | 6.158904971 | 2.73742E-09 |
| GH_A06G1280 | 28.4067681 | 0 | 56.81353619 | Inf | 3.0551E-09 |
| GH_D02G2158 | 80.38447423 | 12.16116789 | 148.6077806 | 3.611155963 | 1.03608E-08 |
| GH_D13G1938 | 169.9087057 | 23.55067004 | 316.2667413 | 3.747301838 | 5.14392E-08 |
| GH_D09G0405 | 406.334901 | 142.7239592 | 669.9458427 | 2.230816933 | 1.07887E-07 |
| GH_A01G2146 | 3610.313817 | 1404.618328 | 5816.009306 | 2.049851412 | 1.14456E-07 |
| GH_A11G1040 | 37.55535679 | 2.971329553 | 72.13938402 | 4.601606575 | 1.20737E-07 |
| GH_A12G1937 | 85.70038234 | 17.52306292 | 153.8777018 | 3.134457311 | 1.47736E-07 |
| GH_D05G0924 | 539.3161465 | 200.0917178 | 878.5405753 | 2.134447466 | 1.78982E-07 |
| GH_D08G0285 | 140.8590545 | 41.81276718 | 239.9053418 | 2.520449852 | 3.75883E-07 |
| GH_D02G0268 | 146.4850849 | 12.53359147 | 280.4365783 | 4.483802758 | 5.82953E-07 |
| GH_A05G0933 | 373.8040718 | 143.5363156 | 604.071828 | 2.073304311 | 7.13404E-07 |
| GH_D13G0063 | 19.41290251 | 0 | 38.82580501 | Inf | 8.08327E-07 |
| GH_D12G0647 | 91.84024099 | 23.61817923 | 160.0623028 | 2.760663916 | 9.37247E-07 |
| GH_A05G1636 | 62.44797256 | 12.68023572 | 112.2157094 | 3.145621187 | 1.05923E-06 |

| | | | | | |
|---|---|---|---|---|---|
| GH_D11G1069 | 185.0950258 | 63.60370617 | 306.5863453 | 2.269110706 | 1.39336E-06 |
| GH_D12G2853 | 241.0902842 | 88.43929684 | 393.7412716 | 2.154488482 | 1.69007E-06 |
| GH_A12G0099 | 135.4136814 | 43.250145 | 227.5772179 | 2.395579266 | 1.84289E-06 |
| GH_D08G0284 | 29.28403058 | 1.95644566 | 56.61161551 | 4.854791149 | 2.38125E-06 |
| GH_A12G0632 | 134.5750409 | 42.94523061 | 226.2048511 | 2.397060046 | 2.51391E-06 |
| GH_D04G0028 | 820.2635783 | 355.543737 | 1284.98342 | 1.853650796 | 3.1357E-06 |
| GH_D02G0264 | 34.72925583 | 3.839569192 | 65.61894248 | 4.095095992 | 3.63316E-06 |
| GH_A05G4218 | 16.77121123 | 0 | 33.54242245 | Inf | 4.68473E-06 |
| GH_D01G2159 | 3455.518182 | 1621.093117 | 5289.943248 | 1.706285282 | 5.10485E-06 |
| GH_D06G0791 | 83.87717632 | 23.90565479 | 143.8486979 | 2.589128334 | 6.12746E-06 |
| GH_A11G3279 | 15.8972625 | 0 | 31.794525 | Inf | 8.25589E-06 |
| GH_A09G0436 | 304.1768871 | 129.835383 | 478.5183912 | 1.881890771 | 9.25296E-06 |
| GH_D12G1645 | 54.34247676 | 11.58040382 | 97.1045497 | 3.067853331 | 9.53448E-06 |
| GH_A01G2058 | 450.3824022 | 196.041562 | 704.7232424 | 1.845897249 | 9.54453E-06 |
| GH_D10G1390 | 90.8937632 | 27.51944466 | 154.2680817 | 2.486916336 | 1.04464E-05 |
| GH_A05G2824 | 67.94006369 | 17.81635143 | 118.0637759 | 2.728292562 | 1.0854E-05 |
| GH_D10G0921 | 99.68173879 | 32.52635494 | 166.8371226 | 2.358759275 | 1.19425E-05 |
| GH_D02G2627 | 255.4849838 | 55.77792353 | 455.1920441 | 3.02870921 | 1.42952E-05 |
| GH_D05G1666 | 27.28138742 | 2.898007426 | 51.66476742 | 4.156047579 | 1.44891E-05 |
| GH_D05G3992 | 17.66702987 | 0.43411982 | 34.89993992 | 6.328987453 | 1.83831E-05 |
| GH_D12G2967 | 367.2064868 | 37.79167607 | 696.6212975 | 4.204234171 | 1.92075E-05 |
| GH_A01G0318 | 54.35904961 | 13.40183111 | 95.31626811 | 2.830292336 | 1.99098E-05 |
| GH_A07G2201 | 567.1933062 | 268.6580768 | 865.7285356 | 1.688143502 | 2.0234E-05 |
| GH_A08G0400 | 188.0413279 | 76.33982519 | 299.7428307 | 1.973217459 | 2.29115E-05 |
| GH_A12G0631 | 149.4154966 | 52.3782871 | 246.4527061 | 2.234270036 | 2.80281E-05 |
| GH_D01G0096 | 393.9069554 | 173.6989236 | 614.1149872 | 1.821919998 | 3.04126E-05 |
| GH_A01G0170 | 28.52515669 | 3.839569192 | 53.21074419 | 3.792701229 | 3.22283E-05 |
| GH_A05G4206 | 330.6556577 | 149.2625751 | 512.0487402 | 1.778428661 | 3.46587E-05 |
| GH_D01G0464 | 134.2956379 | 51.41928651 | 217.1719893 | 2.078456541 | 3.65631E-05 |
| GH_A05G1635 | 521.24618 | 69.8280279 | 972.6643321 | 3.800063881 | 3.77525E-05 |
| GH_D06G0015 | 572.943072 | 267.9364815 | 877.9496625 | 1.712247197 | 4.12194E-05 |

| | | | | | |
|---|---|---|---|---|---|
| GH_D10G0457 | 147.013389 | 60.79064676 | 233.2361312 | 1.939870024 | 4.81628E-05 |
| GH_D09G0614 | 119.4548756 | 44.96918545 | 193.9405658 | 2.10860594 | 4.90665E-05 |
| GH_A05G1223 | 17.97087328 | 1.014883893 | 34.92686268 | 5.104950466 | 5.02289E-05 |
| GH_A01G0105 | 359.7474246 | 170.5425054 | 548.9523437 | 1.686549554 | 5.11852E-05 |
| GH_D06G0779 | 88.90728087 | 30.63160552 | 147.1829562 | 2.264517726 | 5.69297E-05 |
| GH_A12G1799 | 108.9985088 | 41.65449704 | 176.3425206 | 2.081836224 | 6.20545E-05 |
| GH_A04G1203 | 162.5549407 | 64.03201304 | 261.0778683 | 2.027614894 | 6.55389E-05 |
| GH_D12G0052 | 100.9865535 | 38.25486061 | 163.7182463 | 2.097500147 | 6.61876E-05 |
| GH_D07G2193 | 170.1503907 | 74.04583361 | 266.2549477 | 1.846317869 | 6.92309E-05 |
| GH_A01G0106 | 692.7069338 | 349.5125631 | 1035.901304 | 1.567470338 | 7.11161E-05 |
| GH_A07G0767 | 200.2555766 | 90.187402 | 310.3237511 | 1.782776293 | 8.59507E-05 |
| GH_A03G2316 | 28.69520292 | 4.341198195 | 53.04920764 | 3.611166008 | 8.80125E-05 |
| GH_D06G2053 | 27.43255094 | 3.980400503 | 50.88470138 | 3.676246466 | 8.80655E-05 |
| GH_A04G1168 | 58.49107831 | 1.809801405 | 115.1723552 | 5.991819264 | 9.60118E-05 |
| GH_A12G2829 | 338.3519659 | 163.9842045 | 512.7197273 | 1.644613551 | 9.90979E-05 |
| GH_A13G2260 | 106.0958758 | 42.16775193 | 170.0239998 | 2.01152639 | 0.000101847 |
| GH_D12G0107 | 95.59791401 | 36.65921264 | 154.5366154 | 2.075701001 | 0.000103452 |
| GH_D02G0070 | 591.6259523 | 247.6271777 | 935.6247269 | 1.917760327 | 0.000112153 |
| GH_A12G2771 | 94.24983428 | 22.81744877 | 165.6822198 | 2.860209391 | 0.000129893 |
| GH_D01G2237 | 1491.31277 | 799.2299155 | 2183.395625 | 1.449891076 | 0.000140407 |
| GH_A09G2227 | 932.1560423 | 464.0012952 | 1400.310789 | 1.593546321 | 0.000146296 |
| GH_A13G2313 | 8229.607438 | 4542.560847 | 11916.65403 | 1.391401467 | 0.000148286 |
| GH_A06G2325 | 72.40777641 | 24.76226854 | 120.0532843 | 2.277459476 | 0.000149168 |
| GH_A13G2198 | 579.815475 | 305.4639337 | 854.1670163 | 1.483516139 | 0.000151539 |
| GH_D02G1897 | 306.6885238 | 150.8023398 | 462.5747078 | 1.617027573 | 0.000152755 |
| GH_A03G1562 | 162.6071882 | 74.33912212 | 250.8752544 | 1.754776618 | 0.000158511 |
| GH_A02G1968 | 126.5957894 | 54.03563131 | 199.1559475 | 1.881915619 | 0.00016394 |
| GH_A06G0434 | 112.8612389 | 47.30968058 | 178.4127972 | 1.915011776 | 0.000165123 |
| GH_D01G1407 | 343.6021192 | 173.0448374 | 514.1594009 | 1.571069795 | 0.000184838 |
| GH_A06G0804 | 71.34127682 | 24.98223492 | 117.7003187 | 2.236143775 | 0.000186132 |
| GH_A12G1637 | 58.92608676 | 18.82542238 | 99.02675114 | 2.395136074 | 0.000190724 |

| | | | | | |
|---|---|---|---|---|---|
| GH_A01G2389 | 49.41829749 | 14.55754631 | 84.27904867 | 2.533406821 | 0.000190824 |
| GH_A06G1934 | 35.60821345 | 8.474055896 | 62.742371 | 2.888315499 | 0.000228745 |
| GH_D10G2465 | 53.32350645 | 2.604718917 | 104.042294 | 5.319898609 | 0.00023407 |
| GH_A05G4342 | 170.5012845 | 80.63676597 | 260.3658031 | 1.691030291 | 0.000237474 |
| GH_A10G1088 | 25.89969061 | 0 | 51.79938121 | Inf | 0.000245382 |
| GH_A07G1467 | 27.54586831 | 5.215250778 | 49.87648583 | 3.257551288 | 0.000249812 |
| GH_D11G2850 | 102.1597146 | 41.78370246 | 162.5357268 | 1.95974463 | 0.000252483 |
| GH_D01G2467 | 70.06467342 | 25.64213407 | 114.4872128 | 2.15859823 | 0.00025677 |
| GH_A09G1328 | 19.88159623 | 2.537209733 | 37.22598273 | 3.874995248 | 0.000268232 |
| GH_D07G0123 | 411.4386528 | 192.8583251 | 630.0189804 | 1.707853869 | 0.000269032 |
| GH_A03G0087 | 32.53081039 | 1.014883893 | 64.04673689 | 5.979738477 | 0.000283751 |
| GH_A03G2201 | 563.1050988 | 305.0740713 | 821.1361263 | 1.42846184 | 0.000291097 |
| GH_D01G0420 | 186.1410947 | 90.55401264 | 281.7281768 | 1.637453384 | 0.000316197 |
| GH_D07G0549 | 299.0580019 | 18.5495727 | 579.566431 | 4.965516176 | 0.000327724 |
| GH_D13G0429 | 167.3493013 | 79.09118836 | 255.6074143 | 1.692340808 | 0.000332117 |
| GH_A07G0167 | 382.8134173 | 200.9192668 | 564.7075678 | 1.49088805 | 0.000335132 |
| GH_D10G1266 | 47.6345787 | 15.14412333 | 80.12503407 | 2.403495 | 0.000335559 |
| GH_D12G2755 | 471.5949877 | 178.0866254 | 765.10335 | 2.103075469 | 0.000336322 |
| GH_D08G2785 | 44.18340146 | 13.1085426 | 75.25826032 | 2.521342644 | 0.000362665 |
| GH_D12G1794 | 55.38354712 | 19.5644566 | 91.20263765 | 2.220840509 | 0.000409302 |
| GH_D12G1850 | 10.38538347 | 0 | 20.77076694 | Inf | 0.000413988 |
| GH_D05G2976 | 48.41270066 | 15.71907446 | 81.10632686 | 2.367298185 | 0.000434194 |
| GH_D05G1420 | 122.1943537 | 55.7046014 | 188.684106 | 1.760104491 | 0.000436391 |
| GH_D03G1186 | 71.39819187 | 27.89186824 | 114.9045155 | 2.042519018 | 0.000437399 |
| GH_D02G0068 | 748.7551498 | 301.5849946 | 1195.925305 | 1.98749073 | 0.000442987 |
| GH_A06G1737 | 402.9856049 | 208.4191294 | 597.5520804 | 1.519576759 | 0.000508005 |
| GH_D13G1675 | 564.9885609 | 314.2348449 | 815.7422769 | 1.376270256 | 0.000510794 |
| GH_D13G2098 | 315.0084791 | 169.6067565 | 460.4102016 | 1.440726158 | 0.000511765 |
| GH_A07G1847 | 51.24014977 | 17.52887587 | 84.95142368 | 2.276904647 | 0.000549435 |
| GH_A05G0685 | 35.15621413 | 6.884220872 | 63.42820739 | 3.203759279 | 0.000559457 |
| GH_D02G0764 | 34.03605605 | 1.014883893 | 67.05722821 | 6.046006259 | 0.000578392 |

| | | | | | |
|---|---|---|---|---|---|
| GH_D08G0143 | 9.834160869 | 0 | 19.66832174 | Inf | 0.000616171 |
| GH_D05G1166 | 1095.14391 | 625.3320311 | 1564.955789 | 1.323427578 | 0.000621088 |
| GH_A08G2792 | 44.24123739 | 12.82106703 | 75.66140775 | 2.561041284 | 0.000633403 |
| GH_A06G1795 | 370.2069461 | 203.1980657 | 537.2158265 | 1.402615139 | 0.000646983 |
| GH_D01G0497 | 20.42076795 | 3.185482991 | 37.65605292 | 3.563297746 | 0.000692483 |
| GH_A05G1405 | 92.83406398 | 41.14705509 | 144.5210729 | 1.812418785 | 0.000697463 |
| GH_A03G0755 | 103.01204 | 46.94306994 | 159.08101 | 1.760777528 | 0.000698622 |
| GH_D13G1399 | 87.80246033 | 38.24904766 | 137.355873 | 1.844422865 | 0.000706522 |
| GH_A07G0405 | 1383.93956 | 810.1526663 | 1957.726454 | 1.272913494 | 0.000729633 |
| GH_D08G1263 | 140.285133 | 59.76994992 | 220.8003162 | 1.885249999 | 0.000737368 |
| GH_A02G0218 | 102.5881308 | 47.74961334 | 157.4266482 | 1.721118816 | 0.00075434 |
| GH_A11G0963 | 166.6609265 | 83.80481013 | 249.517043 | 1.574033403 | 0.00075507 |
| GH_D13G2310 | 2782.377569 | 1648.517308 | 3916.237831 | 1.248299347 | 0.000776974 |
| GH_A13G2624 | 234.0530519 | 124.6026934 | 343.5034103 | 1.462989168 | 0.000783583 |
| GH_A07G0894 | 1005.016327 | 586.1552937 | 1423.87736 | 1.280470048 | 0.000841668 |
| GH_D10G2037 | 42.46156823 | 14.27588369 | 70.64725277 | 2.307053408 | 0.000856457 |
| GH_D08G2477 | 37.53076499 | 5.288572905 | 69.77295708 | 3.721717603 | 0.000896754 |
| GH_D11G0574 | 361.2393529 | 202.9955382 | 519.4831676 | 1.355628986 | 0.000915525 |
| GH_A09G0437 | 34.60764905 | 10.14302599 | 59.07227211 | 2.541992985 | 0.000921961 |
| GH_D10G1961 | 277.6920727 | 151.1689504 | 404.2151949 | 1.41896171 | 0.00092663 |
| GH_A07G2532 | 535.1816329 | 244.9782014 | 825.3850645 | 1.752413951 | 0.000935077 |
| GH_D09G2123 | 11.38517007 | 0.507441947 | 22.26289819 | 5.455254823 | 0.00094469 |
| GH_D10G1751 | 220.6927012 | 117.4426229 | 323.9427796 | 1.463782908 | 0.00095411 |
| GH_D05G0626 | 73.26969738 | 26.14957602 | 120.3898187 | 2.20285393 | 0.000971099 |
| GH_A13G2531 | 300.6339514 | 160.094565 | 441.1733379 | 1.462421274 | 0.001014188 |
| GH_D08G1324 | 169.7896623 | 87.87015865 | 251.7091659 | 1.518312549 | 0.001023524 |
| GH_A12G2502 | 221.0345255 | 88.95255173 | 353.1164993 | 1.989036335 | 0.001049077 |
| GH_D12G3008 | 1228.797919 | 732.6571259 | 1724.938711 | 1.235335004 | 0.001089412 |
| GH_D07G1005 | 793.7350453 | 361.0277038 | 1226.442387 | 1.76429801 | 0.001109505 |
| GH_D05G3883 | 9.040980416 | 0 | 18.08196083 | Inf | 0.001110451 |
| GH_D02G0279 | 26.99255855 | 6.810898745 | 47.17421835 | 2.792081524 | 0.001149851 |

| | | | | | |
|---|---|---|---|---|---|
| GH_A08G0269 | 12.9328451 | 0 | 25.86569019 | Inf | 0.001169662 |
| GH_D11G0994 | 111.0413439 | 53.96812212 | 168.1145658 | 1.639265335 | 0.001217097 |
| GH_D01G0463 | 636.7710852 | 376.7177136 | 896.8244568 | 1.25134175 | 0.001263609 |
| GH_A04G0036 | 64.57822117 | 26.28459438 | 102.871848 | 1.96855885 | 0.001279151 |
| GH_D09G2160 | 1646.995447 | 967.6809568 | 2326.309936 | 1.265439945 | 0.001287114 |
| GH_A07G2199 | 42.82285868 | 15.65737822 | 69.98833914 | 2.160271915 | 0.001328683 |
| GH_D08G0745 | 25.68318064 | 5.361895032 | 46.00446625 | 3.100959048 | 0.00136242 |
| GH_D09G2230 | 179.999353 | 97.60231659 | 262.3963895 | 1.426760573 | 0.001381556 |
| GH_A06G2198 | 23.52131351 | 5.581861413 | 41.4607656 | 2.892928546 | 0.001391772 |
| GH_D08G2157 | 593.7021735 | 189.8570054 | 997.5473417 | 2.393472078 | 0.001399251 |
| GH_D08G2793 | 33.56610974 | 7.899104765 | 59.23311472 | 2.906642889 | 0.001459665 |
| GH_A06G1679 | 595.1926905 | 352.2697392 | 838.1156419 | 1.250468769 | 0.001477235 |
| GH_D12G0125 | 61.82996634 | 25.78877832 | 97.87115436 | 1.924140285 | 0.001499969 |
| GH_D12G1157 | 93.19841854 | 42.07699097 | 144.3198461 | 1.778166263 | 0.001501355 |
| GH_D08G0415 | 66.5182833 | 28.83343 | 104.2031366 | 1.853584331 | 0.001609122 |
| GH_D07G0132 | 18.65230994 | 3.332127245 | 33.97249264 | 3.349851676 | 0.001633145 |
| GH_A12G2467 | 34.54077501 | 3.986213446 | 65.09533657 | 4.029463324 | 0.001654515 |
| GH_A09G1921 | 16.19130527 | 2.537209733 | 29.8454008 | 3.556193941 | 0.001787268 |
| GH_D11G0199 | 41.35222614 | 14.55754631 | 68.14690597 | 2.226880948 | 0.001818914 |
| GH_D05G3885 | 8.241069274 | 0 | 16.48213855 | Inf | 0.00201898 |
| GH_D01G0636 | 175.8085521 | 63.24872142 | 288.3683829 | 2.188804773 | 0.00202526 |
| GH_D11G3228 | 67.66984773 | 30.20911159 | 105.1305839 | 1.799126767 | 0.002036138 |
| GH_A01G0365 | 483.5220747 | 291.9771546 | 675.0669948 | 1.209175193 | 0.002041016 |
| GH_A05G2116 | 123.1163976 | 64.19028319 | 182.042512 | 1.503848568 | 0.002047551 |
| GH_D05G1410 | 279.8612878 | 162.942502 | 396.7800735 | 1.283976608 | 0.002060235 |
| GH_D12G2360 | 15.84165638 | 2.537209733 | 29.14610303 | 3.521988314 | 0.002081519 |
| GH_A12G0222 | 678.3235952 | 411.8139098 | 944.8332806 | 1.198067222 | 0.002091724 |
| GH_D05G2890 | 504.5005268 | 301.2496948 | 707.7513587 | 1.232282834 | 0.002120705 |
| GH_D02G0069 | 352.9477224 | 184.6636857 | 521.231759 | 1.497024804 | 0.002121081 |
| GH_D05G4004 | 21.2836098 | 1.375681586 | 41.19153802 | 4.904129506 | 0.002130102 |
| GH_A05G1477 | 186.9869903 | 104.7623871 | 269.2115935 | 1.361619702 | 0.002216509 |

| | | | | | |
|---|---|---|---|---|---|
| GH_A12G2726 | 522.1283249 | 257.8050814 | 786.4515684 | 1.609077224 | 0.002218346 |
| GH_D11G0831 | 124.2194932 | 64.39281074 | 184.0461756 | 1.515096242 | 0.002260029 |
| GH_D10G0075 | 16.82568944 | 2.824685299 | 30.82669359 | 3.448018108 | 0.002262786 |
| GH_A01G0644 | 194.6070713 | 109.6901624 | 279.5239803 | 1.349537914 | 0.002288853 |
| GH_A04G1209 | 163.8030491 | 88.42767095 | 239.1784273 | 1.435517476 | 0.00229829 |
| GH_A05G0629 | 106.4781667 | 53.82147787 | 159.1348555 | 1.563995954 | 0.002302004 |
| GH_A13G1786 | 71.76776617 | 33.76701816 | 109.7685142 | 1.700777602 | 0.002435912 |
| GH_A12G1309 | 375.3337734 | 131.0934851 | 619.5740616 | 2.240680757 | 0.002477602 |
| GH_A05G0912 | 130.1320044 | 69.84546673 | 190.418542 | 1.446935581 | 0.002542646 |
| GH_D10G2466 | 2000.810021 | 595.4524064 | 3406.167637 | 2.516091334 | 0.002550106 |
| GH_A09G0560 | 63.1841465 | 27.95937742 | 98.40891558 | 1.81545679 | 0.002565792 |
| GH_A13G1128 | 291.9994966 | 156.0408423 | 427.9581508 | 1.455546034 | 0.002585319 |
| GH_D01G0087 | 29.4918602 | 9.201464224 | 49.78225618 | 2.435696256 | 0.00260787 |
| GH_A07G2040 | 26.70682004 | 7.825782638 | 45.58785744 | 2.542342662 | 0.002649908 |
| GH_A13G2341 | 67.76405697 | 31.15067335 | 104.3774406 | 1.744474678 | 0.00265621 |
| GH_D13G1867 | 1064.760897 | 519.8489741 | 1609.672821 | 1.630603019 | 0.00266323 |
| GH_A12G1965 | 442.1484058 | 218.6913609 | 665.6054506 | 1.605771018 | 0.002722907 |
| GH_A07G0431 | 273.0253166 | 160.3878535 | 385.6627797 | 1.265775031 | 0.002740858 |
| GH_A10G2580 | 327.1991214 | 187.091375 | 467.3068678 | 1.320627189 | 0.002748996 |
| GH_A09G2228 | 5545.424394 | 3527.340333 | 7563.508454 | 1.100474827 | 0.002813777 |
| GH_D03G1875 | 35.51385911 | 12.53359147 | 58.49412675 | 2.2224919 | 0.002855284 |
| GH_A01G0634 | 120.5894519 | 63.45124897 | 177.7276548 | 1.485947718 | 0.002907687 |
| GH_A05G0217 | 63.19407142 | 26.80947516 | 99.57866768 | 1.893093736 | 0.002941214 |
| GH_A07G1418 | 323.4210474 | 182.0880315 | 464.7540634 | 1.35183138 | 0.00299716 |
| GH_D13G0473 | 374.5074874 | 221.0622414 | 527.9527334 | 1.255956148 | 0.003095668 |
| GH_A05G1798 | 1167.55255 | 726.280347 | 1608.824754 | 1.147408738 | 0.003127655 |
| GH_D12G1628 | 296.147801 | 127.3679286 | 464.9276734 | 1.868004249 | 0.00316426 |
| GH_A13G1255 | 170.6019096 | 94.91264966 | 246.2911696 | 1.37569262 | 0.003206056 |
| GH_A13G1721 | 309.3889375 | 186.0358005 | 432.7420745 | 1.217927119 | 0.003230364 |
| GH_D08G1860 | 179.3181274 | 80.20264615 | 278.4336086 | 1.795611622 | 0.003259078 |
| GH_D11G0071 | 44.50852047 | 18.26209714 | 70.7549438 | 1.953978508 | 0.003291667 |

| | | | | | |
|---|---|---|---|---|---|
| GH_A10G2381 | 809.2323896 | 66.54015806 | 1551.924621 | 4.543689382 | 0.003310331 |
| GH_D05G1256 | 261.3871877 | 155.8383148 | 366.9360606 | 1.235478711 | 0.003386042 |
| GH_D05G0300 | 26.87074989 | 8.113258203 | 45.62824158 | 2.491573749 | 0.003404735 |
| GH_A05G3214 | 320.8683105 | 193.8557702 | 447.8808509 | 1.208131307 | 0.003464794 |
| GH_A01G2403 | 3786.399909 | 2339.368405 | 5233.431414 | 1.161638115 | 0.003494859 |
| GH_D06G0407 | 123.3539189 | 60.42984906 | 186.2779888 | 1.624123967 | 0.003543921 |
| GH_A11G0501 | 502.1901442 | 308.2456823 | 696.134606 | 1.175285609 | 0.003570673 |
| GH_D05G1667 | 53.92897427 | 20.5677146 | 87.29023393 | 2.085438757 | 0.003591231 |
| GH_D01G0347 | 424.9427007 | 250.9651179 | 598.9202835 | 1.254877137 | 0.003707717 |
| GH_D03G1481 | 177.1345992 | 101.5652783 | 252.7039201 | 1.315040767 | 0.003725583 |
| GH_D13G2221 | 79.6011636 | 40.14379708 | 119.0585301 | 1.568422 | 0.00375882 |
| GH_D09G1551 | 636.7723732 | 400.3010151 | 873.2437313 | 1.125299107 | 0.003809491 |
| GH_D06G0265 | 661.32409 | 405.8202548 | 916.8279251 | 1.175810114 | 0.003869604 |
| GH_A01G1556 | 582.7201939 | 368.5311332 | 796.9092546 | 1.112628949 | 0.004100969 |
| GH_D10G1788 | 141.0874052 | 79.4019157 | 202.7728948 | 1.352619096 | 0.004109613 |
| GH_A06G1514 | 68.45019951 | 33.03379689 | 103.8666021 | 1.652717129 | 0.004158206 |
| GH_D04G1888 | 156.7910219 | 88.58594109 | 224.9961028 | 1.344750351 | 0.004174759 |
| GH_D05G2889 | 40.63737984 | 16.15319428 | 65.12156539 | 2.011315895 | 0.004238489 |
| GH_D06G2043 | 410.0861246 | 58.56191823 | 761.6103309 | 3.701018333 | 0.004241521 |
| GH_A08G2800 | 19.75266799 | 4.927775212 | 34.57756076 | 2.810827747 | 0.004261006 |
| GH_D01G0423 | 68.28371015 | 32.74050838 | 103.8269119 | 1.66503181 | 0.004353401 |
| GH_A09G1614 | 13.20237882 | 1.883123532 | 24.52163411 | 3.702855574 | 0.004393891 |
| GH_A12G2141 | 497.0375445 | 197.9828149 | 796.0922741 | 2.007560453 | 0.004395842 |
| GH_D06G1764 | 423.142736 | 263.0914081 | 583.194064 | 1.148411901 | 0.004476652 |
| GH_A09G1993 | 27.79551611 | 8.981497842 | 46.60953437 | 2.375597132 | 0.004483901 |
| GH_D05G3925 | 10.5468996 | 0.941561766 | 20.15223744 | 4.419740477 | 0.004500733 |
| GH_A01G1052 | 370.638639 | 194.5156693 | 546.7616087 | 1.491025569 | 0.004671299 |
| GH_D05G3871 | 42.59472485 | 17.01562098 | 68.17382873 | 2.002358206 | 0.004704041 |
| GH_A13G1859 | 29.80885885 | 7.899104765 | 51.71861294 | 2.710922522 | 0.00470829 |
| GH_D05G2446 | 704.8968864 | 444.798957 | 964.9948158 | 1.117367787 | 0.004751192 |
| GH_D12G2518 | 325.5922696 | 158.668813 | 492.5157262 | 1.634151202 | 0.004773436 |

| | | | | | |
|---|---|---|---|---|---|
| GH_A12G0384 | 154.6520957 | 89.26909201 | 220.0350994 | 1.30150102 | 0.00487502 |
| GH_A04G0136 | 37.66940487 | 10.2838573 | 65.05495243 | 2.661277392 | 0.004989497 |
| GH_A13G0880 | 90.09132647 | 47.30386763 | 132.8787853 | 1.49008074 | 0.005066092 |
| GH_A05G0554 | 1057.299464 | 684.3768188 | 1430.222109 | 1.063376412 | 0.005089092 |
| GH_A11G1467 | 23.09697079 | 6.884220872 | 39.30972072 | 2.513520824 | 0.005106679 |
| GH_D06G1976 | 25.12146235 | 6.52342318 | 43.71950152 | 2.744575826 | 0.005194976 |
| GH_D12G0152 | 82.62506155 | 26.1612019 | 139.0889212 | 2.410506782 | 0.005256142 |
| GH_scaffold7261_objG0001 | 1608.380455 | 1039.54364 | 2177.21727 | 1.066535061 | 0.005266943 |
| GH_A10G2374 | 151.0118756 | 18.53213387 | 283.4916174 | 3.935205162 | 0.005284824 |
| GH_A11G2867 | 1553.600281 | 1016.671233 | 2090.529328 | 1.040015061 | 0.005298589 |
| GH_D07G1809 | 1253.411671 | 819.7439929 | 1687.07935 | 1.041282501 | 0.005356094 |
| GH_D12G0645 | 379.5188242 | 235.0377029 | 523.9999455 | 1.15667446 | 0.005368646 |
| GH_D12G0236 | 756.7333827 | 489.7051255 | 1023.76164 | 1.063894654 | 0.005479813 |
| GH_D02G1765 | 15.04076244 | 2.898007426 | 27.18351745 | 3.229598951 | 0.005527302 |
| GH_A10G0215 | 31.62166787 | 11.5920297 | 51.65130604 | 2.155671633 | 0.005528284 |
| GH_D05G1807 | 128.0594309 | 71.92530487 | 184.1935569 | 1.35665126 | 0.005542065 |
| GH_A01G2197 | 122.9443418 | 69.47885609 | 176.4098275 | 1.344285027 | 0.005550527 |
| GH_A03G0829 | 117.7167316 | 66.65998373 | 168.7734794 | 1.34019535 | 0.005715081 |
| GH_A09G1423 | 172.9972848 | 101.007766 | 244.9868037 | 1.278237821 | 0.005717751 |
| GH_D05G2891 | 1380.283706 | 101.58853 | 2658.978883 | 4.710062892 | 0.006021149 |
| GH_D07G0093 | 19.50940077 | 4.347011139 | 34.67179041 | 2.99566664 | 0.006127203 |
| GH_D11G0520 | 1086.001679 | 703.197749 | 1468.805609 | 1.062641116 | 0.006161284 |
| GH_A12G1258 | 372.8148053 | 237.1850503 | 508.4445603 | 1.100077394 | 0.0062737 |
| GH_D05G1409 | 573.8185096 | 372.2240582 | 775.4129611 | 1.058793545 | 0.006275943 |
| GH_D13G2202 | 13.60082038 | 2.317243352 | 24.8843974 | 3.424759985 | 0.006293825 |
| GH_A08G1035 | 288.9203801 | 181.1138382 | 396.7269219 | 1.131249521 | 0.006438837 |
| GH_D02G2198 | 1029.905859 | 679.5071731 | 1380.304545 | 1.022425927 | 0.00653104 |
| GH_A10G2377 | 19.51009582 | 5.141928651 | 33.87826299 | 2.71997841 | 0.006580183 |
| GH_A07G2362 | 202.3045432 | 124.3326567 | 280.2764297 | 1.172645145 | 0.006713865 |
| GH_A09G1606 | 865.6624399 | 570.9342814 | 1160.390598 | 1.023213916 | 0.006758036 |
| GH_A13G2220 | 21.17491243 | 5.863524035 | 36.48630082 | 2.637514986 | 0.006784196 |

| | | | | | |
|---|---|---|---|---|---|
| GH_A13G1347 | 788.3790286 | 520.2482162 | 1056.509841 | 1.022034187 | 0.006788838 |
| GH_D02G0593 | 113.2721299 | 64.26360531 | 162.2806545 | 1.3364172 | 0.006808583 |
| GH_A10G1748 | 115.933604 | 66.2933731 | 165.5738348 | 1.32053814 | 0.006882516 |
| GH_D05G2063 | 542.4884008 | 343.6164075 | 741.3603941 | 1.109376118 | 0.006888632 |
| GH_D07G0434 | 208.5848727 | 109.9776379 | 307.1921074 | 1.481930944 | 0.007010318 |
| GH_A07G0534 | 13.43891534 | 2.463887606 | 24.41394307 | 3.30869697 | 0.007063626 |
| GH_A03G0107 | 227.8096321 | 137.5797845 | 318.0394797 | 1.208937364 | 0.007069283 |
| GH_A02G2027 | 44.82371498 | 19.69947496 | 69.947955 | 1.8281247 | 0.007083271 |
| GH_A05G0349 | 62.58693453 | 31.44396186 | 93.7299072 | 1.575726449 | 0.007109145 |
| GH_D10G0227 | 179.47708 | 106.3848537 | 252.5693063 | 1.24738656 | 0.007114599 |
| GH_D13G0367 | 36.39314641 | 14.92415695 | 57.86213588 | 1.954970141 | 0.007134681 |
| GH_D12G0212 | 61.66441583 | 30.20329865 | 93.12553302 | 1.624470656 | 0.007156945 |
| GH_A05G2640 | 143.689485 | 84.39138715 | 202.9875829 | 1.266223806 | 0.007200542 |
| GH_D13G0189 | 244.0423506 | 128.9054471 | 359.179254 | 1.478390794 | 0.0073113 |
| GH_A02G0099 | 72.36837221 | 38.1140293 | 106.6227151 | 1.484120787 | 0.007328801 |
| GH_D02G0125 | 59.49395939 | 29.70748259 | 89.2804362 | 1.58751772 | 0.007546955 |
| GH_D10G0244 | 78.77747726 | 42.38190537 | 115.1730492 | 1.442282808 | 0.008235112 |
| GH_A08G2039 | 29.31593869 | 10.71797712 | 47.91390025 | 2.160411614 | 0.008235378 |
| GH_A05G1166 | 616.756518 | 404.6073357 | 828.9057003 | 1.034685507 | 0.008333355 |
| GH_A08G0989 | 219.3346367 | 136.1830972 | 302.4861763 | 1.151321562 | 0.008416146 |
| GH_A11G2254 | 47.24429359 | 21.5825985 | 72.90598869 | 1.756168754 | 0.008433764 |
| GH_A07G2432 | 10.78114397 | 1.302359459 | 20.25992847 | 3.95942948 | 0.008441058 |
| GH_A02G1569 | 309.6167479 | 197.8361707 | 421.3973251 | 1.090874936 | 0.00849572 |
| GH_D11G1129 | 194.1084546 | 118.7892397 | 269.4276695 | 1.181493861 | 0.008508897 |
| GH_A10G1542 | 68.41298606 | 36.01093938 | 100.8150327 | 1.485203639 | 0.008574815 |
| GH_D12G2002 | 149.5910078 | 90.34567214 | 208.8363434 | 1.208845405 | 0.008756968 |
| GH_A13G0724 | 196.2783979 | 119.5667184 | 272.9900774 | 1.191032644 | 0.008803111 |
| GH_A10G2271 | 534.3999656 | 349.0994489 | 719.7004822 | 1.043758546 | 0.008844912 |
| GH_A11G1099 | 234.161891 | 146.0212088 | 322.3025732 | 1.142237778 | 0.008886679 |
| GH_A07G2044 | 132.6978739 | 76.50972122 | 188.8860266 | 1.303801007 | 0.008929434 |
| GH_D07G0193 | 42.55575393 | 19.34449021 | 65.76701764 | 1.76544154 | 0.008976885 |

| | | | | | |
|---|---|---|---|---|---|
| GH_D13G1747 | 18.77749157 | 5.141928651 | 32.4130545 | 2.656193485 | 0.009003701 |
| GH_D13G2148 | 783.2552441 | 518.055686 | 1048.454802 | 1.017085582 | 0.009061052 |
| GH_A05G2772 | 187.9559797 | 116.7152145 | 259.1967449 | 1.151054963 | 0.009079844 |
| GH_D07G0176 | 187.423549 | 114.0639921 | 260.7831058 | 1.19300698 | 0.009165152 |
| GH_A07G1719 | 1043.239214 | 620.8696866 | 1465.608741 | 1.239137612 | 0.009206076 |
| GH_A07G2146 | 187.7148692 | 113.6915685 | 261.7381698 | 1.202999065 | 0.009302146 |
| GH_D11G3377 | 103.9072387 | 59.62330567 | 148.1911718 | 1.313511235 | 0.009308649 |
| GH_A08G2322 | 47.93121664 | 22.96990597 | 72.89252731 | 1.666025971 | 0.009330421 |
| GH_A05G1960 | 85.10916861 | 46.72310356 | 123.4952337 | 1.402247349 | 0.009385915 |
| GH_D03G0878 | 387.5710206 | 251.786854 | 523.3551872 | 1.055587437 | 0.009415922 |
| GH_D11G2333 | 41.51859081 | 18.91037039 | 64.12681122 | 1.761750048 | 0.009571465 |
| GH_D12G1963 | 149.2014229 | 90.41318133 | 207.9896644 | 1.201906815 | 0.009604048 |
| GH_D12G2773 | 253.2003783 | 158.9714813 | 347.4292754 | 1.127951349 | 0.009625271 |
| GH_A07G0332 | 41.14193885 | 13.03522047 | 69.24865723 | 2.409371113 | 0.009650574 |
| GH_D13G1765 | 15.99349656 | 1.375681586 | 30.61131153 | 4.475846369 | 0.009662933 |
| GH_D01G2158 | 272.5217348 | 175.1769921 | 369.8664776 | 1.078191247 | 0.009671223 |
| GH_D09G2370 | 168.8147456 | 103.8825216 | 233.7469696 | 1.169994722 | 0.009751312 |
| GH_D05G0560 | 104.8962811 | 59.67918896 | 150.1133732 | 1.330752674 | 0.009764183 |
| GH_D10G0242 | 193.6236035 | 121.4230234 | 265.8241836 | 1.13043036 | 0.009911123 |
| GH_D01G0057 | 65.52477615 | 31.44396186 | 99.60559043 | 1.663443712 | 0.009972188 |
| GH_A08G2599 | 105.4112907 | 59.67337602 | 151.1492054 | 1.34081409 | 0.010009201 |
| GH_A13G1455 | 600.5306834 | 312.1666327 | 888.894734 | 1.509696244 | 0.010048105 |
| GH_A13G1148 | 64.78594965 | 33.46210376 | 96.10979553 | 1.522155328 | 0.0101139 |
| GH_A06G1711 | 6.130408526 | 0 | 12.26081705 | Inf | 0.010165161 |
| GH_D05G0354 | 44.78738048 | 20.5677146 | 69.00704635 | 1.746362187 | 0.01017037 |
| GH_A01G1593 | 577.938451 | 220.1962479 | 935.6806542 | 2.087226339 | 0.01023901 |
| GH_A12G2220 | 6654.839548 | 3954.52428 | 9355.154815 | 1.242257376 | 0.010309921 |
| GH_A11G0438 | 360.9493979 | 236.0118962 | 485.8868997 | 1.041760955 | 0.01031457 |
| GH_A08G1541 | 421.1363575 | 270.0896417 | 572.1830734 | 1.083038508 | 0.010367793 |
| GH_A10G2234 | 6.069832321 | 0 | 12.13966464 | Inf | 0.010415104 |
| GH_A12G2614 | 13.69635939 | 2.898007426 | 24.49471135 | 3.079337094 | 0.01054086 |

| | | | | | |
|---|---|---|---|---|---|
| GH_A09G2399 | 2127.335223 | 1402.936016 | 2851.73443 | 1.023390422 | 0.010658436 |
| GH_A11G1527 | 78.94052545 | 43.09768781 | 114.7833631 | 1.413231175 | 0.010724875 |
| GH_D04G0921 | 52.29089338 | 25.93542258 | 78.64636419 | 1.600456195 | 0.01073614 |
| GH_D08G2611 | 13.52579999 | 2.678041044 | 24.37355894 | 3.186066946 | 0.010845834 |
| GH_A08G0200 | 16.96592957 | 4.059535573 | 29.87232356 | 2.879422867 | 0.01091226 |
| GH_A04G1284 | 243.5575221 | 149.4731617 | 337.6418824 | 1.175607404 | 0.010967983 |
| GH_A12G2704 | 277.4073344 | 176.9228511 | 377.8918178 | 1.094852885 | 0.011064636 |
| GH_A11G1534 | 115.8697916 | 68.53148138 | 163.2081018 | 1.251873898 | 0.011075665 |
| GH_A09G1317 | 26.6911053 | 9.703093227 | 43.67911738 | 2.170427065 | 0.011137396 |
| GH_A06G0856 | 84.32067362 | 47.23054551 | 121.4108017 | 1.362104678 | 0.011281611 |
| GH_A13G0433 | 201.1696599 | 126.0284454 | 276.3108744 | 1.132542946 | 0.011281708 |
| GH_A08G0953 | 28.38032981 | 11.01126563 | 45.74939399 | 2.054772332 | 0.011289845 |
| GH_D11G0268 | 261.1995395 | 164.7268055 | 357.6722735 | 1.118562946 | 0.011420165 |
| GH_D12G2400 | 516.9655036 | 335.8862733 | 698.044734 | 1.055346656 | 0.01145482 |
| GH_D07G0073 | 31.50816661 | 13.2609998 | 49.75533342 | 1.907661631 | 0.011582804 |
| GH_D02G0800 | 241.0078384 | 154.0669579 | 327.948719 | 1.089912754 | 0.011638143 |
| GH_A02G0220 | 67.33275774 | 36.4450592 | 98.22045628 | 1.43030028 | 0.011665165 |
| GH_D02G1891 | 411.2335804 | 269.5088776 | 552.9582832 | 1.036837847 | 0.011676909 |
| GH_D11G1426 | 267.0139215 | 172.5373955 | 361.4904475 | 1.067048441 | 0.012006148 |
| GH_D01G0787 | 45.01237929 | 21.51508931 | 68.50966927 | 1.670958791 | 0.012006583 |
| GH_D06G0403 | 27.46961888 | 10.87043432 | 44.06880343 | 2.019348142 | 0.012025719 |
| GH_A12G0052 | 60.40268431 | 31.51147105 | 89.29389758 | 1.502684479 | 0.012056873 |
| GH_A07G1092 | 14.08976851 | 2.824685299 | 25.35485173 | 3.166099788 | 0.012124341 |
| GH_A12G2940 | 542.1803243 | 235.9850776 | 848.375571 | 1.846007444 | 0.012248555 |
| GH_D01G0611 | 16.12874305 | 4.200366884 | 28.05711921 | 2.739779635 | 0.012434719 |
| GH_D06G2181 | 92.35783578 | 53.75978163 | 130.9558899 | 1.284481765 | 0.012621495 |
| GH_A13G1935 | 30.99311232 | 12.67442278 | 49.31180185 | 1.960012925 | 0.012651676 |
| GH_D05G1951 | 170.8034907 | 106.6222589 | 234.9847225 | 1.140058311 | 0.012843737 |
| GH_D11G2399 | 93.16911843 | 53.67483362 | 132.6634032 | 1.305452722 | 0.012876569 |
| GH_A13G2553 | 135.7145765 | 83.16234982 | 188.2668032 | 1.178776206 | 0.012912231 |
| GH_A06G2016 | 27.98074754 | 0.868239639 | 55.09325543 | 5.987638614 | 0.013057521 |

| | | | | | |
|---|---|---|---|---|---|
| GH_D09G1316 | 40.93717049 | 19.05120171 | 62.82313928 | 1.721414034 | 0.013091117 |
| GH_A02G2053 | 109.7752872 | 64.97357481 | 154.5769995 | 1.250400679 | 0.013116804 |
| GH_D01G1168 | 56.1697448 | 26.00293176 | 86.33655783 | 1.731297282 | 0.013209163 |
| GH_D09G1022 | 277.2429875 | 159.4927953 | 394.9931798 | 1.308336488 | 0.013409097 |
| GH_A07G0903 | 33.30217568 | 14.56335926 | 52.0409921 | 1.837305292 | 0.013480342 |
| GH_D05G2614 | 13.57559588 | 1.809801405 | 25.34139035 | 3.80759238 | 0.013515528 |
| GH_A06G2120 | 62.3930893 | 26.78041044 | 98.00576815 | 1.87168859 | 0.013596724 |
| GH_D09G2481 | 187.1489647 | 119.1616633 | 255.1362661 | 1.098347817 | 0.013710633 |
| GH_A07G0122 | 166.0345066 | 103.6451164 | 228.4238969 | 1.140061447 | 0.013857961 |
| GH_A07G0768 | 133.3424006 | 82.00663462 | 184.6781665 | 1.171200776 | 0.01390427 |
| GH_D08G0499 | 331.3097379 | 113.8265869 | 548.792889 | 2.269424216 | 0.013940438 |
| GH_A09G1745 | 93.48975469 | 53.5898856 | 133.3896238 | 1.315613804 | 0.014049798 |
| GH_A13G2031 | 202.8051008 | 130.1729289 | 275.4372726 | 1.081294347 | 0.014128086 |
| GH_D12G2617 | 22.07189735 | 7.899104765 | 36.24468994 | 2.198008586 | 0.014246949 |
| GH_A06G1849 | 92.44625249 | 53.74815574 | 131.1443492 | 1.286868488 | 0.014274884 |
| GH_A10G0004 | 73.46105285 | 40.48715595 | 106.4349498 | 1.394435754 | 0.014296612 |
| GH_A13G1708 | 177.6314548 | 112.1983074 | 243.0646022 | 1.115288896 | 0.014320249 |
| GH_D09G1728 | 43.4546394 | 21.00764736 | 65.90163143 | 1.649399576 | 0.014429171 |
| GH_A01G2008 | 28.31805535 | 11.51870758 | 45.11740312 | 1.969705179 | 0.014467948 |
| GH_D11G3547 | 32.92402733 | 14.19674862 | 51.65130604 | 1.863244272 | 0.01455362 |
| GH_D02G0213 | 169.6791321 | 106.3906666 | 232.9675975 | 1.130757718 | 0.014693089 |
