# Supplementary material for: AtZAT10/STZ1 improves drought tolerance and increases fiber yield in cotton
Source: Front Plant Sci. 2024 Oct 21;15:1464828. doi: 10.3389/fpls.2024.1464828 (PMC11532130; doi:10.3389/fpls.2024.1464828)
Supplement: Supplementary Appendix S2 — 106 differentially expressed genes of AtSTZ1-overexpressing transgenic cotton lines in comparison with the wild type following drought stress. [file DataSheet2.pdf]

Gene ID	KEGG	GO
GH_A01G0048	K15397	GO:0016747;GO:0006633;GO:0016021
GH_A01G0439	-	GO:0030247;GO:0006468;GO:0005524;GO:0016021;GO:0004674
GH_A01G0440	-	GO:0030247;GO:0006468;GO:0005524;GO:0016021;GO:0004674
GH_A01G0512	-	GO:0030246
GH_A01G0588	-	GO:0016021;GO:0005634;GO:0003677
GH_A01G0644	-	GO:0006355;GO:0003677;GO:0005634
GH_A01G0989	K07760	GO:0006468;GO:0005524;GO:0004674
GH_A01G1298	-	GO:0016758;GO:0008152
GH_A01G2021	-	GO:0005975;GO:0004553
GH_A01G2189	-	GO:0006468;GO:0005524;GO:0016021;GO:0004674
GH_A01G2389	K12657	GO:0005737;GO:0016310;GO:0006561;GO:0004349;GO:0004350;GO:0055114
GH_A02G0017	-	GO:0055085;GO:0016021
GH_A02G1771	-	-
GH_A02G1960	-	GO:0005886;GO:0004672;GO:0006468;GO:0005524;GO:0016021
GH_A03G0090	-	-
GH_A03G0575	K14424	GO:0006633;GO:0016491;GO:0005506;GO:0016021;GO:0055114
GH_A03G0715	K06674	GO:0000796;GO:0007076;GO:0005524;GO:0005634
GH_A03G2049	K07374	GO:0005737;GO:0005200;GO:0005525;GO:0007017;GO:0003924;GO:0005874

GH_A04G0041	-	GO:0006468;GO:0005524;GO:0016021;GO:0004674
GH_A04G0142	-	-
GH_A04G1341	K16290	GO:0008234;GO:0006508
GH_A04G1412	K07374	GO:0005737;GO:0005200;GO:0005525;GO:0007017;GO:0003924;GO:0005874
GH_A05G0051	K01904	GO:0016874;GO:0008152
GH_A05G0304	K02959	GO:0005840;GO:0003735;GO:0006412
GH_A05G0355	-	GO:0043086;GO:0004857
GH_A05G0374	K01051	GO:0042545;GO:0005618;GO:0030599;GO:0045330;GO:0004857;GO:0043086;GO:0045490
GH_A05G1647	-	-
GH_A05G2040	-	-
GH_A05G2319	-	GO:0005509
GH_A05G3870	K03798	GO:0004222;GO:0005524;GO:0006508;GO:0016020
GH_A06G0211	K13051	GO:0016787
GH_A07G0164	K08908	GO:0046872;GO:0009765;GO:0018298;GO:0016168;GO:0016021;GO:0009522;GO:0009535
GH_A07G0314	K00131	GO:0016620;GO:0055114
GH_A07G0896	-	GO:0016021;GO:0055114

GH_A07G1015	K00224	GO:0009941;GO:0005773;GO:0016491;GO:0005886;GO:0008270;GO:0009535;GO:0055114
GH_A07G1346	-	GO:0046872;GO:0005737
GH_A07G2345	-	-
GH_A08G1487	-	GO:0016021;GO:0007165
GH_A08G1493	-	-
GH_A08G1956	-	GO:0009505;GO:0006508;GO:0004190
GH_A09G0082	K16189	GO:0046983
GH_A09G1779	K13993	-
GH_A09G1932	K01056	GO:0004045
GH_A10G0275	-	GO:0016021
GH_A10G0285	-	-
GH_A10G1398	K17285	GO:0008430
GH_A10G2234	-	-
GH_A11G1292	-	-
GH_A11G3279	-	-
GH_A12G1538	-	GO:0006950
GH_A12G2502	-	GO:0003824;GO:0008152
GH_A12G2843	-	GO:0004672;GO:0006468;GO:0005524;GO:0016021
GH_A12G2848	-	GO:0016021

GH_A13G0724	K01626	GO:0003849;GO:0009073;GO:0009534
GH_A13G0727	-	-
GH_A13G1255	K01738	GO:0004124;GO:0006535;GO:0016740
GH_D01G0611	-	-
GH_D01G2168	-	-
GH_D02G0727	-	GO:0016021
GH_D02G0760	-	-
GH_D02G0959	K01723	GO:0020037;GO:0016705;GO:0004497;GO:0005506;GO:0055114
GH_D03G0641	K09873	GO:0016021;GO:0009506;GO:0015250;GO:0009941;GO:0009705;GO:0009505;GO:0072489;GO:0015200;GO:0006833;GO:0000326;GO:0042807;GO:0005794;GO:0005886
GH_D03G1091	-	GO:0046983
GH_D03G1445	-	-
GH_D04G0279	K14487	-
GH_D04G2031	-	GO:0020037;GO:0016021;GO:0016705;GO:0004497;GO:0005506;GO:0055114
GH_D05G3556	-	-
GH_D05G3668	-	GO:0006952;GO:0043531
GH_D05G3946	K12160	GO:0005634
GH_D06G0128	-	GO:0016021;GO:0009535;GO:0009512

GH_D06G0129	K00472	-
GH_D06G1018	K09286	GO:0006355;GO:0003700;GO:0003677;GO:0005634
GH_D06G1175	-	-
GH_D06G2094	-	GO:0016021;GO:0006810;GO:0005215
GH_D08G0405	K09422	GO:0003677
GH_D08G0628	-	GO:0016021
GH_D08G0771	K11251	GO:0046982;GO:0000786;GO:0005634;GO:0003677
GH_D08G1015	K07964	GO:0016798;GO:0005975;GO:0016020
GH_D08G1545	-	GO:0051087
GH_D08G2330	K13508	GO:0016746;GO:0016021;GO:0008152
GH_D08G2550	-	-
GH_D09G1371	-	GO:0020037;GO:0016021;GO:0016705;GO:0004497;GO:0005506;GO:0055114
GH_D09G1833	K16903	GO:0048467;GO:0009958;GO:0008483;GO:0042742;GO:0009723;GO:0010087;GO:0048527;GO:0043562;GO:0005789;GO:0016846;GO:0010078
GH_D09G2227	K01183	GO:0004568;GO:0005975;GO:0006032;GO:0008061;GO:0016998
GH_D09G2516	-	GO:0016874
GH_D10G0658	K13248	GO:0016311;GO:0016791

GH_D10G1882	K19589	GO:0003676;GO:0006479;GO:0008276
GH_D10G1944	-	GO:0016874
GH_D11G0978	-	GO:0016021;GO:0004650;GO:0005975
GH_D11G1490	-	-
GH_D11G1649	-	GO:0016760;GO:0071555;GO:0030244;GO:0016021
GH_D11G3096	-	GO:0042626;GO:0055085;GO:0005524;GO:0016021
GH_D11G3499	K21777	GO:0005634
GH_D12G1863	-	-
GH_D12G1866	K15925	GO:0030246;GO:0005975;GO:0004553
GH_D12G1911	-	GO:0042626;GO:0005886;GO:0055085;GO:0005524;GO:0016021
GH_D12G2674	K00430	GO:0046872;GO:0098869;GO:0006979;GO:0042744;GO:0020037;GO:0004601;GO:0005576;GO:0055114
GH_D12G2894	K09422	GO:0008152;GO:0003677;GO:0003824
GH_D12G2948	K02183	GO:0005509
GH_D13G0313	-	GO:0006355;GO:0003677;GO:0005634
GH_D13G0636	K00814	GO:0030170;GO:0009058;GO:0008483
GH_D13G2234	K02357	GO:0003746;GO:0005622;GO:0006414
GH_D13G2562	K09873	GO:0016021;GO:0006810;GO:0005215

GH_D13G2599	K01904	GO:0016874;GO:0008152
GH_scaffold418_objG0001	K20659	GO:0016866
GH_scaffold7261_objG0001	-	GO:0008146
