# Supplementary material for: AtZAT10/STZ1 improves drought tolerance and increases fiber yield in cotton
Source: Front Plant Sci. 2024 Oct 21;15:1464828. doi: 10.3389/fpls.2024.1464828 (PMC11532130; doi:10.3389/fpls.2024.1464828)
Supplement: Supplementary file 3 [file DataSheet1.docx]

***Supplementary Material***

**
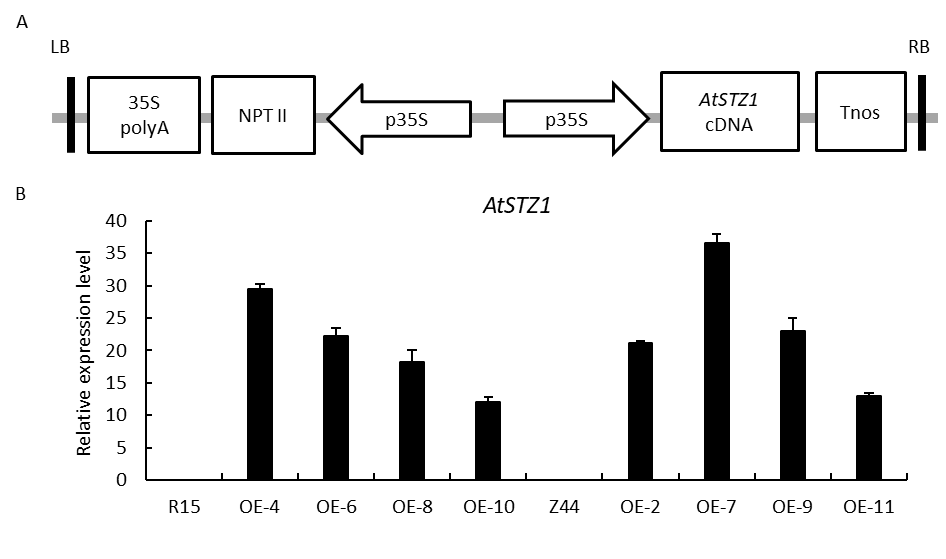
**

**Supplementary Figure S1** Overexpression vector and the expression level of *AtSTZ1* in transgenic cotton plants. (A) Schematic representation of the T-DNA region of the *AtSTZ1*-overexpressing vector. LB, left border; RB, right border; 35S polyA, cauliflower mosaic virus (CaMV) 35S polyA; NPT II, neomycin phosphotransferase gene; p35S, CaMV 35S promoter; Tnos, 3'-termination signal of nopaline synthase. (B) *AtSTZ1* transcript levels in 5-day-old seedlings from independent T_3_ transgenic lines determined by quantitative RT‒PCR. *GhUBI1* was used as an internal control. The values are the means ± SDs of independent triplicate assays. R15 and Z44, wild-type cotton (R15 and Zhongmian 44). OE-4, -6, -8, -10, *AtSTZ1* transgenic cotton lines 4, 6, 8 and 10, generated using R15 as explants; OE-2, -7, -9 and -11, *AtSTZ1* transgenic cotton lines 2, 7, 9 and 11 generated using Z44 as explants.


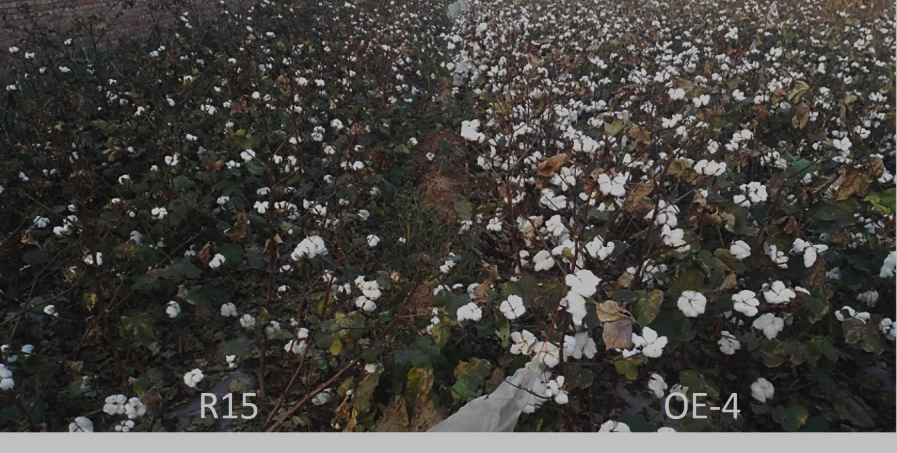


**Supplementary Figure S2** Boll growth of *AtSTZ1* transgenic cotton lines in the field during the stage of maturity. R15: wild-type receptor control R15; OE-4: *AtSTZ1*-overexpressing transgenic cotton line 4 generated using R15 as explants.

**Supplementary Table S1** The primers utilized in the study.

| Gene | Sequence (5’ to 3’) | Purpose |
| --- | --- | --- |
| *STZ1*OE  *STZ1*  *GhLRK10*  *GhTMK1-L*  *GhRGI3*  *GhBAK1*  *GH_A02G1960*  *GhPERK2*  *GhTIP2-1*  *GhTIP1-3*  *GhCALM1*  *GhHSP18.1*  *GhPOD5*  *GhLIN1*  *GhDREB3*  *GhZFN56L*  *GhBHLH72*  *GhCML29*  *GhBLH6*  *GhNAC72L*  *GhMYB3*  *GhMYB62* | P1: 5′-CTTGGATCATGGCGGCTCGAGGCTCTT-3′  P2: 5′-CTTGCGGCCGCTTAAAGTTGAAGTTTG-3′  RT P1:5′-ATGGCGGCTCGAGGCTCTTACAT-3′  RT P2: 5′-TTAAAGTTGAAGTTTGACCGGA-3′  RT P1:5′- GGAGTGGGTCGAGGTATTGA-3′  RT P2:5′-CGGAGGTATCTCAAGGAGCTC-3′  RTP1:5′-GGGAGTGGTTCTGCAAGCAG -3′  RTP2: 5′-CAGCGTGGCCCATGTCTGG-3′  RTP1:5′-GTGGCGGTAAGGGAGGAGCTG-3′  RTP2: 5′- GCAGTTCGATGATCCATCCGC -3′  RTP1: 5′-GCCAAGGAGGGGTACAGGA-3′  RTP2: 5′-GGACGGCCATTGCAGTTACCC-3′  RTP1: 5′- CCTGCCGATCTGTCTGGCC-3′  RTP2: 5′-CACTGTCGGTGCTGCTCCG-3′  RTP1: 5′-ATGGCTTCTCCTATTAACTTC-3′  RTP2: 5′-GCAGAGTAGAATGGAGATTGG-3′  RTP1: 5′-ATGGCAGGAATCGCCTTTGG-3′  RTP2: 5′-CATTGGACAATGGTGCATGG-3′  RTP1: 5′-ATGCCGATTTCTCGAATTGC-3′  RTP2: 5′-GGGGTTCAGGACGCACCGT-3′  RTP1: 5′-ATGGTGGACCAACTCACCGA-3′  RTP2: 5′-TCACTTTGCCACCATCATCC-3′  RTP1: 5′-ATGGCCATGATTCCACGAATG-3′  RTP2: 5′-ACTGGACATGGATCTGAATGG-3′  RTP1: 5′-GAGACCAAGCAAGCTAAGTC-3′  RTP2: 5′-CGCGCGCAACCCCGTATCAG-3′  RTP1:5′-ATGGGAAGGGCTCCTTGTTG-3′  RTP2: 5′-ATGGGAAGGGCTCCTTGTTG-3′  RTP1:5′- ATGGCTGAGCTACAACATTC-3′  RTP2: 5′- AGCAAGCCTTGTTCAAAACC-3′  RTP1:5′- GCACCAACGGGTTGGCCTCA-3′  RTP2: 5′- GAGGTTGCCAGTGTGGTCGG-3′  RTP1: 5′-CCAAGAGGTGGTGTTGGAGAG-3′  RTP2: 5′-GAGCAGCCGCCGCGAAGCGCG-3′  RTP1: 5′-ATGGCTCAACTAGGGTCGTC-3′  RTP2: 5′-ATGAGTCTGAAGTCCTCC-3′  RTP1:5′-GCAGATTGTGGTATCCTCG-3′  RTP2: 5′-GGAACTCAACCTGTGCTGCC-3′  RTP1:5′-GCCGGGTCCGGGTACTGGA-3′  RTP2: 5′-TGTCCACTCGGTACCAGCTC-3′  RTP1:5′-ATGGGAAGGGCTCCTTGTTG-3′  RTP2: 5′-CCATGGTCGGCGGCGTAGGC-3′  RTP1:5′-CATCATCGTCGTCGTCATCA-3′  RTP2: 5′-AGTGGCAATCCGCGGCGGGG-3′ | vector construction  qRT-PCR  qRT-PCR  qRT-PCR  qRT-PCR  qRT-PCR  qRT-PCR  qRT-PCR  qRT-PCR  qRT-PCR  qRT-PCR  qRT-PCR  qRT-PCR  qRT-PCR  qRT-PCR  qRT-PCR  qRT-PCR  qRT-PCR  qRT-PCR  qRT-PCR  qRT-PCR  qRT-PCR |

**Supplementary Table S2** Statistical analysis of DEGs in different samples.

| Control | Case | Up-regulated Genes | Down-regulated Genes | Total DEGs |
| --- | --- | --- | --- | --- |
| M_Z44 | D_Z44 | 2954 | 2906 | 5860 |
| M_R15 | D_R15 | 2981 | 2824 | 5805 |
| M_OE_7﹠M_OE_9﹠M_OE_4 | D_OE_7﹠D_OE_9﹠D_OE_4 | 204 | 188 | 392 |
| M_OE_7﹠M_OE_9 | D_OE_7﹠D_OE_9 | 262 | 205 | 467 |
| M_OE_4﹠M_OE_7 | D_OE_4﹠D_OE_7 | 252 | 234 | 486 |
| M_OE_4﹠M_OE_9 | D_OE_4﹠D_OE_9 | 241 | 233 | 474 |
| D_ Z44 ﹠D_R15  D_ Z44﹠D_OE_7  D_ Z44﹠D_OE_7  D_ Z44﹠D_OE_9 | D_OE_4﹠D_OE_7﹠D_OE_9  D_ Z44﹠D_OE_9  D_ R15﹠D_OE_4  D_ R15﹠D_OE_4 | 60  484  153  164 | 56  351  125  131 | 106  835  278  295 |
| M_Z44 | M_OE_7 | 501 | 478 | 979 |
| M_Z44 | M_OE_9 | 1289 | 972 | 2261 |
| M_OE_7 | D_OE_7 | 699 | 726 | 1425 |
| M_OE_9 | D_OE_9 | 776 | 1968 | 2744 |
| D_Z44 | D_OE_7 | 3201 | 956 | 4157 |
| D_Z44 | D_ OE_9 | 1693 | 943 | 2636 |
| M_R15 | M_OE_4 | 307 | 1405 | 1712 |
| D_R15 | D_ OE_4 | 868 | 1233 | 2101 |
| M_OE_4 | D_OE_4 | 697 | 356 | 1053 |

Control: control group; Case: experimental group (treatment group); Upregulated genes: upregulated genes in the case group compared with the control group; Downregulated genes: downregulated genes in the case group compared with the control group; Total DEGs: DEGs between the case group and the control group. R15, Z44: Wild-type receptor control R15 and Zhongmian 44; OE-4, *AtSTZ1-*overexpressing transgenic cotton lines 4 generated using R15 as explants; OE-7, -9: *AtSTZ1*-overexpressing transgenic cotton lines 7 and 9 generated using Zhongmian 44 as explants. M: mock, under normal growth conditions; D: under drought treatment.

**Supplementary Table S3** Majorly upregulated genes in *AtSTZ1* transgenic cotton lines following drought stress according to the RNA-Seq data.

| Gene name | Gene ID | Type | Function annotation |
| --- | --- | --- | --- |
| *GhLRK10* | GH_A01G0439 | kinase | PR5-like receptor kinase |
| *GhTMK1-L*  *GhRGI3* | GH_A01G2189  GH_A04G0041 | kinase  kinase | receptor protein kinase TMK1-like  LRR receptor-like serine/threonine-protein kinase |
| *GhBAK1* | GH_A12G2843 | kinase | LRR receptor-like serine/threonine-protein kinase |
| *GhLRR60* | GH_A02G1960 | kinase | LRR receptor-like serine/threonine-protein kinase |
| *GhPERK2* | GH_D02G0727 | kinase | proline-rich receptor-like protein kinase |
| *GhTIP2-1* | GH_D03G0641 | Functional gene | aquaporin |
| *GhTIP1-3* | GH_D13G2562 | Functional gene | aquaporin |
| *GhCAMP1* | GH_D12G2948 | Ca^2+^signaling gene | calmodulin-like protein 1 |
| *GhCML29* | GH_A05G2319 | Ca^2+^signaling gene | calcium-binding protein CML29 |
| *GhLIN1* | GH_D09G2516 | Functional gene | E3 ubiquitin-protein ligase |
| *GhPOD5* | GH_D12G2674 | Antioxidant enzymes activities | peroxidase 5 |
| *GhDREB3L* | GH_D06G1018 | Transcription factor | dehydration-responsive element-binding protein 3-like |
| *GhZFN56L* | GH_A07G1346 | Transcription factor | zinc finger CCCH domain-containing protein 56-like isoform X1 |
| *GhBHLH72* | GH_D03G1091 | Transcription factor | PIF7-like isoform X2 |
| *GhBLH6* | GH_D13G0313 | Transcription factor | BEL1-like homeodomain protein 6 |
| *GhNAC72L* | GH_A01G0644 | Transcription factor | NAC domain-containing protein 72-like |
| *GhMYB3* | GH_D08G0405 | Transcription factor | myb-related protein 308-like |
| *MYB62* | GH_D12G2894 | Transcription factor | MYB108-like |
| *HSP18.1* | GH_A09G1779 | Heat stress protein | 18.1 kDa class I heat shock protein |
| *GhHSP18.1L* | GH_D09G0373 | Heat stress protein | 18.1 kDa class I heat shock protein-like [*Gossypium raimondii*] |
| *GhHSP70L* | GH_D09G2300 | Heat stress protein | Heat shock 70 kda protein-like |
| *GhHSPL* | GH_A05G0923 | Heat stress protein | Low molecular weight heat shock protein |
| *GhHSP17.3* | GH_D05G0915 | Heat stress protein | 17.3 kDa class I heat shock protein [*Gossypium hirsutum*] |
| *GhHSP22* | GH_D08G1971 | Heat stress protein | Heat shock 22 kda protein |
| *GhHSP90L* | GH_D03G1673 | Heat stress protein | Heat shock protein 90-1-like [*Gossypium raimondii*] |
| *GhHSP8L* | GH_A09G2360 | Heat stress protein | Heat shock 70 kda protein 8-like [*Gossypium hirsutum*] |
| *GhHSP2L* | GH_A07G0271 | Heat stress protein | 16.9 kDa class I heat shock protein 2-like [*Gossypium hirsutum*] |
| *GhHSP10* | GH_A02G0345 | Heat stress protein | 18.1 kDa class I heat shock protein-like [*Gossypium raimondii*] |
